# Lactate Activates the E2F Pathway to Promote Cell Motility by Up-Regulating Microtubule Modulating Genes

**DOI:** 10.3390/cancers11030274

**Published:** 2019-02-26

**Authors:** Yi-deun Jung, Jung Hee Cho, Seulki Park, Minho Kang, Seung-jin Park, Dong Hee Choi, Moonkyung Jeong, Kyung Chan Park, Young Il Yeom, Dong Chul Lee

**Affiliations:** 1 Immunotherapy Research Center, Korea Research Institute of Bioscience and Biotechnology (KRIBB), Daejeon 34141, Korea; yideunj@kribb.re.kr (Y.-d.J.); cjh@kribb.re.kr (J.H.C.); krr1013@kribb.re.kr (S.P.); 2 Department of Functional Genomics, University of Science and Technology (UST), Daejeon 34113, Korea; prosium@kribb.re.kr (S.-j.P.); kpark@kribb.re.kr (K.C.P.); 3 Personalized Genomic Medicine Research Center, Korea Research Institute of Bioscience and Biotechnology (KRIBB), Daejeon 34141, Korea; mhkang@kribb.re.kr (M.K.); mkjeong@kribb.re.kr (M.J.); 4 Laboratory Animal Resource Center, Korea Research Institute of Bioscience and Biotechnology (KRIBB), Daejeon 34141, Korea; heedc@kribb.re.kr

**Keywords:** Lactate, metastasis, cell motility, microtubule, E2F pathway, kinesin family genes

## Abstract

Excess lactate production due to enhanced aerobic glycolysis is characteristic of malignant cancers, which is also intimately associated with poor cancer prognoses. Although tumor-associated lactate contributes to all major steps in carcinogenesis, its action mechanism remains obscure. To understand the molecular mechanism of the lactate-induced tumor metastatic process, we identified an array of lactate-responsive genes via transcriptome analysis of a metformin-induced hyper-glycolytic liver cancer model. Gene set enrichment analysis suggested E2F-RB pathway as the dominant regulator of the lactate-induced gene expression. We experimentally verified that lactate indeed activates E2F-mediated transcription by promoting E2F1 protein accumulation through a posttranscriptional mechanism. Literature-based analysis of target pathways potentially modulated by 136 top-ranked genes indicated that genes functioning in cell-cell or cell-matrix communications dominate the lactate-induced gene expression. Especially, those regulating microtubule functions, including a group of kinesin family members, were significantly up-regulated in lactate- and E2F1-dependent manners. Depletion of E2F1 or kinesins (KIF2C, KIF18B, KIF20A) led to deformation of microtubule structures, impairing cell motility as much as the deficit in lactate production. These results indicate that E2F pathway activation by tumor-associated lactate and subsequent transcriptional activation of microtubule functions play crucial roles in tumor metastasis, providing mechanistic clues to cell motility-directed anti-cancer strategies.

## 1. Introduction

The pathophysiological conditions of tumor microenvironment are closely connected to various stages of tumor development as well as the response to cancer therapeutic approaches. Although oncology researches regarding tumor microenvironment have intensively focused on the intricate networks of extracellular matrix molecules and hypoxia [1,2], the cellular metabolism in tumor microenvironment is beginning to receive significant attention only recently. Lactate is a soluble metabolite present in abundance within tumor microenvironment [3,4]. Increased glycolytic flux associated with malignant tumor cells leads to enhanced lactate production even in the presence of oxygen. This phenomenon, termed “aerobic glycolysis” or “Warburg effect”, is a critical part of the deregulated cellular energetics in cancers cells, which has been recognized as the hallmark of cancer recently [5,6]. Accordingly, the lactate-enriched microenvironment has been regarded as the potential driver of tumor growth and metastasis and thus as a favorable target for cancer therapy.

Lactate is known to contribute to all major steps of carcinogenesis, including angiogenesis, immune escape, cell migration, and metastasis [4]. Importantly, tumor metastasis is the main cause of cancer-related death, and therefore, its prevention is one of the major goals of cancer therapy. In many types of solid cancers, lactate concentration is highly correlated with tumor metastasis [7,8]. In its roles in cell motility regulation, lactate functions as a key element in increasing endothelial cell migration [9]. In glioma cells, TGF-β2 that acts as a key regulator of cell migration was induced by lactate [10]. Also, exogenous lactate has a significant effect on the lung metastasis of breast cancer cells without increasing tumor growth [11]. Lactate accumulation actively enhances the degree of tumor malignancy [12]. Thus, it has been well established that lactate plays a key role in tumor metastasis; however, the mechanisms underlying the lactate-mediated metastatic process are still poorly understood.

Tumor metastasis undergoes a multistep development that includes migration and invasion of cancer cells [13]. These processes require the involvement of a wide array of cellular mechanisms led by cytoskeleton dynamics including microtubules and actin filaments [13]. Microtubules have emerged as important regulators of cell migration machinery, controlling force generation, substrate adhesion and signaling pathways [14]. Many of these functions are mediated or modulated by kinesins, microtubule motor proteins that transport a variety of molecules and organelles within the cell [14]. Among the 45 kinds of kinesins expressed in human cells, KIF2C, KIF4, and KIF3A are best known for their contribution to the regulation of microtubule dynamics [15,16,17]. Recent reports indicated that inhibition of these kinesins results in decreased cell migration [18,19]. Consistently, alteration of microtubule dynamics has been proven effective in controlling tumor metastasis [20].

Here, we established a lactate-enriched environment using hepatocellular carcinoma cell lines carrying metformin-induced mitochondrial respiratory dysfunction to understand the molecular mechanisms of lactate-medicated tumor metastatic process. We identified genes responsible for the lactate-mediated enhancement of cell motility, including a group of kinesin family members, which have been shown to regulate cell motility by targeting microtubules. Lactate-enriched environment enhanced microtubule dynamics by transcriptionally up-regulating kinesins via E2F1 pathway. These results provide a novel mechanistic link between lactate-mediated tumor metastasis and microtubule dynamics, and support the role of kinesins as the potential therapeutic target of tumor metastasis.

## 2. Results

### 2.1. Identification of the Genes Associated with Lactate-Driven Cell Motility in a Metformin-Induced Glycolytic Cell Model

To investigate the role of lactate in the regulation of tumor cell motility, we modeled a lactate-enriched environment using metformin, a known inhibitor of complex I of the mitochondrial electron transport chain. Transient metformin treatment in HepG2 cells clearly upregulated the extra- and intra-cellular lactate concentration (Figure S1A,B) by rendering the cells hyper-glycolytic (Figure S1C,D). In parallel, metformin-treated HepG2 cells exhibited significantly enhanced migration and invasion compared to parental cells (Figure S1E). The mitochondrial dysfunction-associated glycolytic phenotypes were repeatedly observed as HepG2 cells transiently treated with rotenone, another inhibitor of the mitochondrial electron transport chain complex I, exhibited enhanced lactate production, upregulated glycolysis rates and increased migratory properties (Figure S1F). To gain mechanistic insights into the lactate-induced pro-migratory phenotypes, we established a cell model that acquired a resistance to metformin while producing lactate at a high level by selecting HepG2 cells that survived a chronic exposure to metformin for more than 5 months (HepG2/metR). These cells assumed flat, large, and irregular shapes, a morphology that is characteristic of mesenchymal cells in contrast to parental cells (Figure S1G), and appeared to retain improved mitochondrial functions as they exhibited increased oxygen consumption rates in the presence of metformin (Figure S1H). 

**Figure 1:**
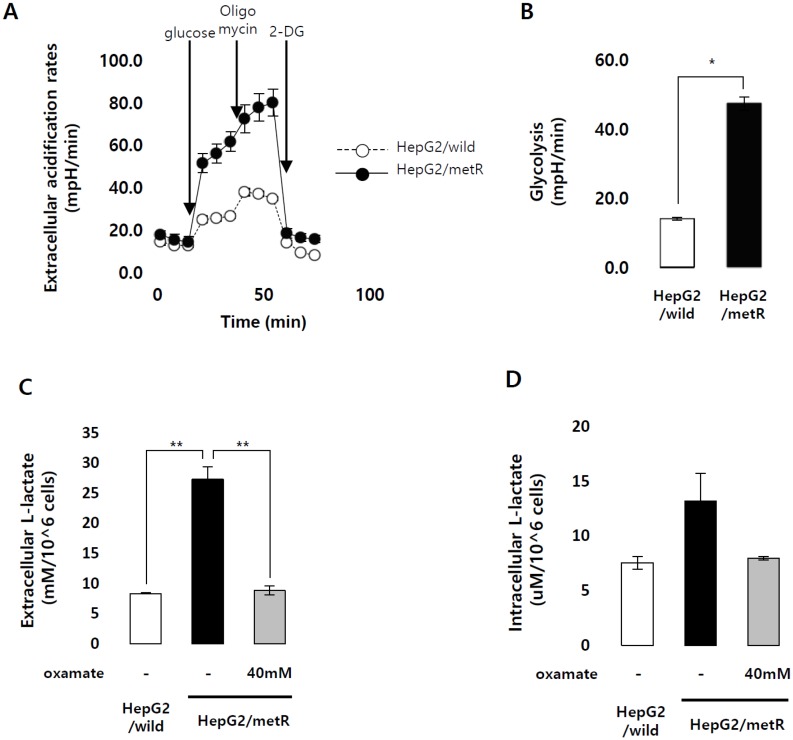

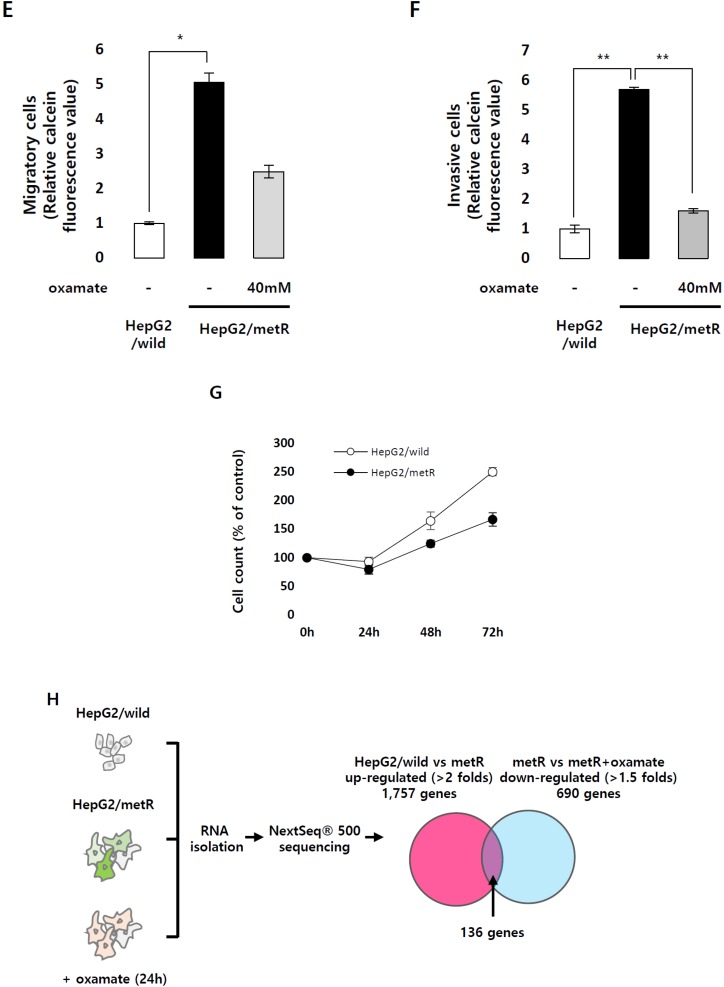
Identification of genes differentially expressed in a lactate-enriched microenvironment. (**A**) Kinetic extracellular acidification rates (ECAR) response of parental HepG2 and HepG2/metR cells to glucose (20 mM), oligomycin (1 µM), and 2-DG (50 mM), respectively. ECAR values were normalized by the BCA protein assay. A representative experiment out of at least three trials is shown. (**B**) The glycolytic activity was measured by quantitatively estimating the conversion of glucose to pyruvate in HepG2 and HepG2/metR cells after the addition of a saturating amount of glucose to a given cell. *p*-value was assessed by Student’s *t*-test using data from triplicate experiments. * *p* < 0.05 and ** *p* < 0.01 vs. control. (**C**) Extracellular lactate production by HepG2, HepG2/metR, and oxamate-treated HepG2/metR cells. Results are mean ± SD of three experiments. The *p*-value was assessed by Student’s *t*-test. * *p* < 0.05 and ** *p* < 0.01 vs. control. (**D**) Intracellular lactate production by HepG2, HepG2/metR, and oxamate-treated HepG2/metR cells. Results are mean ± SD of three experiments. The *p*-value was assessed by Student’s *t*-test. (**E**,**F**). Migratory and invasive properties of HepG2, HepG2/metR, and oxamate-treated HepG2/metR cells as represented by relative calcein-AM fluorescence values. Results from three independent experiments are shown as mean ± SD. * *p* < 0.05 and ** *p* < 0.01 vs. control by Student’s *t*-test. (**G**) Growth of HepG2 and HepG2/metR cells were quantitatively estimated by cell counting during indicated time period. Results from three independent experiments are shown as means ± SD. (**H**) Schematic illustration of the RNA-sequencing strategy to profile lactate-induced gene expression.

In HepG2/metR cells, glycolysis rates were increased by more than 3-fold compared with parental cells (Figure 1A,B), and consequently, lactate production was also highly enhanced (Figure 1C,D). They also exhibited a highly increased motility compared to control HepG2 cells (Figure 1E,F, Figure S1I). To clarify the relationship between the lactate level and cell motility in the HepG2/metR model, we treated the cells with oxamate, an inhibitor of lactate dehydrogenase, and found that it significantly decreased both extra- and intra-cellular lactate levels (Figure 1E,F). In parallel, the increase in cell motility was abrogated by oxamate treatment (Figure S1I). We also verified the pro-migratory effects of lactate by treating HepG2 or Huh1 cells with exogenously provided l-lactate (Figure S1J,K). These data confirm that lactate plays a key regulatory role in cell motility. However, the growth rate of HepG2/metR cells was slightly slower than that of parental cells (Figure 1G), indicating that the increased motility of HepG2/metR cells may be one hallmark of the lactate-enriched microenvironment.

To determine the genes potentially responsible for the lactate-mediated cell motility regulation in hepatocellular carcinoma, we performed RNA-seq analyses on parental HepG2, HepG2/metR and oxamate-treated HepG2/metR cells. Using a 2-fold change cut-off value in transcriptome, we selected 1757 genes significantly up-regulated in HepG2/metR vs parental HepG2 cells. 690 genes were down-regulated by oxamate treatment in HepG2/metR cells. Eventually, we selected 136 genes that are common in the two gene sets, which may directly respond to lactate signaling (Figure 1H, Table S1).

### 2.2. Lactate Up-Regulates the Expression of Genes Modulating Microtubules Including a Group of Kinesin Family Genes

**Figure 2:**
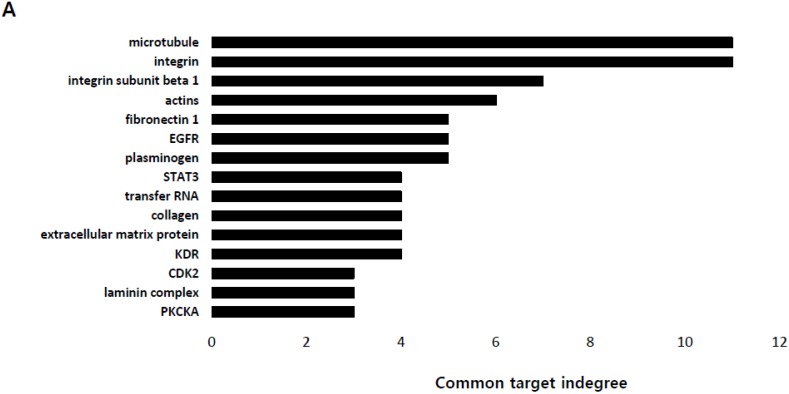

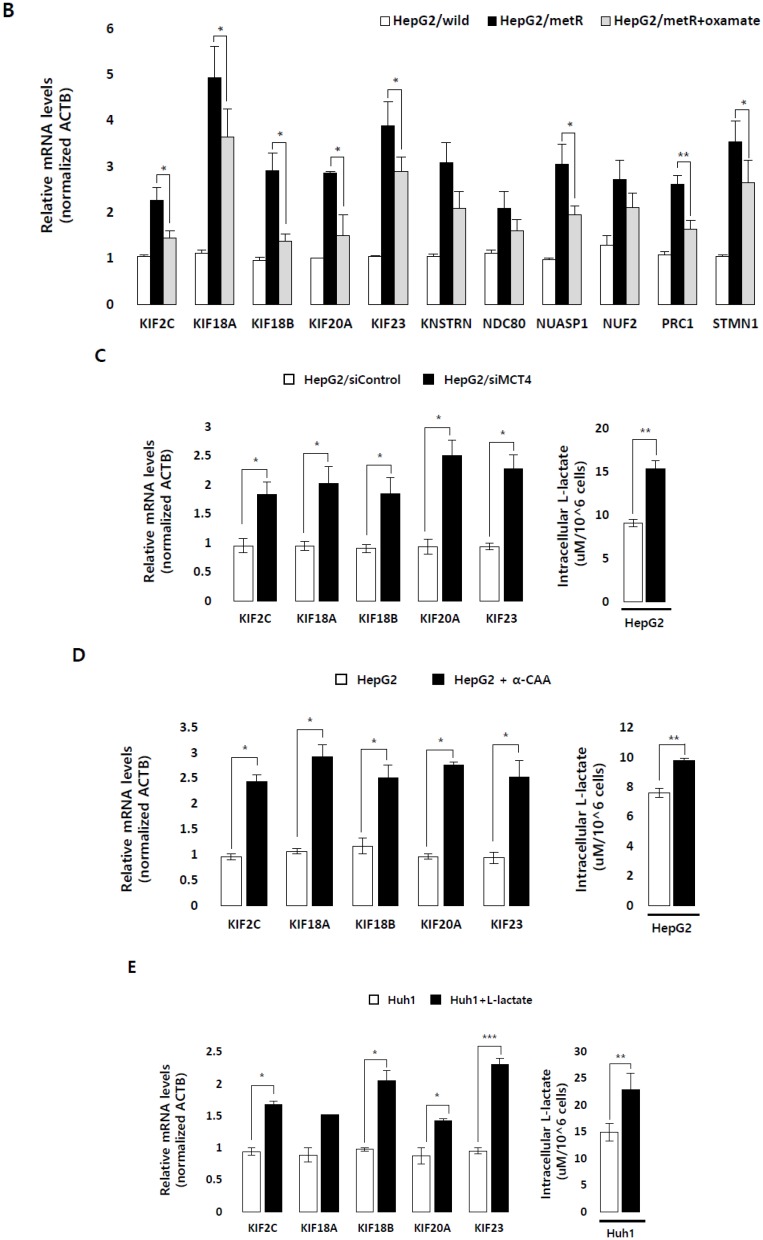
Lactate-enriched microenvironment up-regulates the expression of genes modulating cell-cell or cell-matrix communications. (**A**) Pathways potentially regulated by 136 top-ranked lactate-responsive genes were identified in silico through a literature mining process provided by Pathway Studio program. (**B**) mRNA expression pattern of the eleven lactate-responsive genes related to ‘microtubule functions’, including 5 kinesin family members. mRNA levels were determined by quantitative RT-PCR (qRT-PCR). (**C**–**E**) Lactate-dependent expression of kinesin family genes were verified by qRT-PCR in HepG2 cells (C) depleted of MCT4 expression by siRNA or (**D**) treated with a lactate transport inhibitor, α-CAA (2.5 mM), or (E) in Huh1 cells treated with exogenous l-lactate (30 mM). Changes in intracellular lactate concentration due to the depletion or inhibition of MCT4 or exogenous l-lactate treatment are shown on the right in each panel. All results are from three independent experiments and shown as mean ± SD. For qRT-PCR the expression level of transcripts for each gene was normalized by Ct-value of ACTB. The *p*-value was assessed by Student’s *t*-test. * *p* < 0.05 and ** *p* < 0.01 vs. control.

Bioinformatic analysis of the biological pathways that can be functionally modulated by the 136 lactate-responsive genes revealed a dominance of pathways related to cell-cell or cell-matrix communications, with the highest enrichment in the microtubule and integrin pathways (Figure 2A). Microtubules are one of the major components of cytoskeleton, playing essential roles in cell division, migration, and polarization [21]. Thus this analysis raises a possibility that regulation of microtubules might be one of the major processes driving lactate-induced cell motility. To validate this hypothesis, we first examined the expression pattern of eleven genes constituting the ‘microtubule’ term, and confirmed that their mRNA expression levels are largely dependent on lactate concentration (Figure 2B). Interestingly, five of the 11 genes were kinesin family members, i.e., KIF2C, KIF18A, KIF18B, KIF20A and KIF23, and this led us to focus on kinesins in subsequent studies for the molecular mechanism of lactate-driven cell motility (see below). Importantly, all of them consistently showed a significant lactate dependence of mRNA expression. Thus, genetic knockdown (siRNA) or pharmacological inhibition (α-CAA) of the membrane transporter MCT4, which is responsible for the export of excess intracellular lactate, caused their up-regulation in HepG2 cells with concomitant increase in lactate concentration (Figure 2C,D and Figure S2A). The lactate-dependent kinesin expression was equally confirmed by exogenous l-lactate treatment in Huh1 cells (Figure 2E). Conversely, suppression of lactate production by inhibition of LDHA via oxamate treatment in Huh7 cells resulted in down-regulation of kinesins (Figure S2B). Taken together, these results suggest that kinesin family genes may play an important role in lactate-mediated cell motility process.

### 2.3. E2F1 Is a Pivotal Upstream Regulator of Lactate-Induced Gene Expression

**Figure 3:**
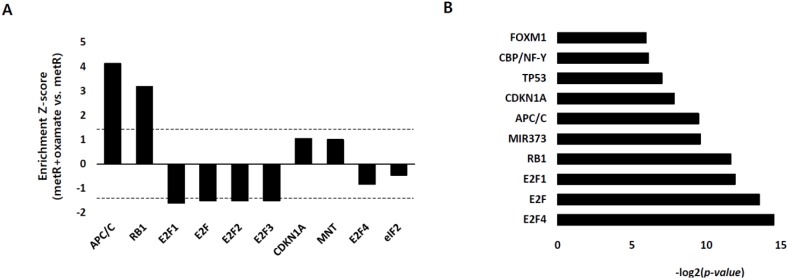

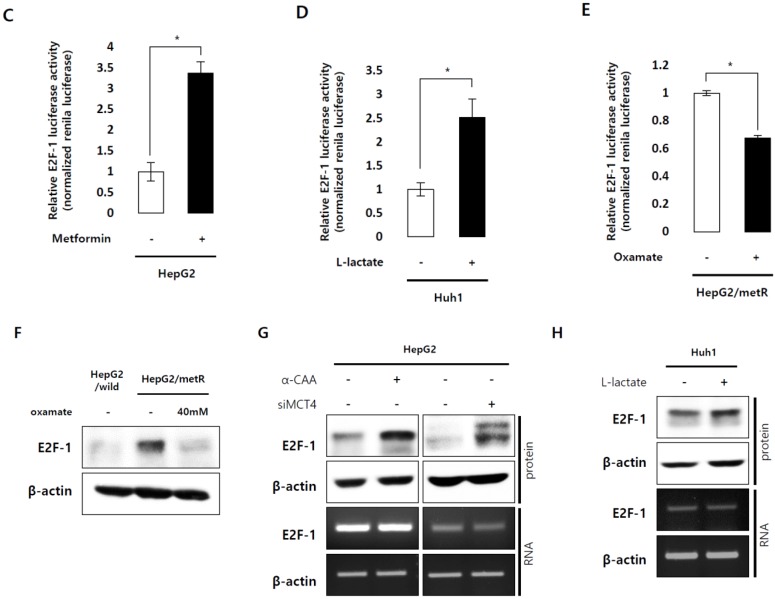
Role of E2F1 pathway in lactate-induced gene expression and its regulation by lactate. (**A**) Profiling of upstream regulators of lactate-induced gene expression. Transcriptome data for HepG2/metR cells with or without oxamate treatment were subject to a sub-network enrichment analysis using Pathway Studio program, and the activation score (Z-score) of putative upstream regulators was quantitatively estimated. Dotted lines represent a statistical significance of *p* < 0.05 in both directions. (**B**) Upstream regulators predicted for the eleven lactate-responsive genes related to ‘microtubule functions’ through the literature mining process of Pathway Studio. (**C**–**E**). Lactate-dependent transcription activity of E2F1 was measured by E2F1-driven luciferase reporter assays in (**C**) metformin (10 mM)-treated HepG2 cells, (**D**) exogenous l-lactate (50 mM)-treated Huh1 cells, and (**E**) oxamate (40 mM)-treated HepG2/metR cells. The luciferase activity values were normalized by *renilla* luciferase values. All results from three independent experiments are shown as mean ± SD. The *p*-value was assessed by Student’s *t*-test. * *p* < 0.05 and ** *p* < 0.01 vs. control. (**F**–**H**). The protein and mRNA expression of E2F1 gene in various lactate-enriched microenvironments including (**F**) oxamate (40 mM)-treated HepG2/metR cells, (**G**) pharmacological inhibition of lactate transporter or siRNA-mediated depletion of MCT4 in HepG2 cells, and (**H**) exogenous l-lactate treatment in Huh1 cells.

We next profiled dominant upstream regulators of the lactate-induced gene expression through a sub-network enrichment analysis (Pathway Studio, Elsevier, Amsterdam, The Netherlands), where the regulatory activity (Z-score) of putative upstream regulators was quantitatively estimated from the transcriptome data for HepG2/metR cells with or without oxamate treatment. The results for top-ranked regulators indicated that different E2F family members exhibited significantly reduced transcriptional activities upon oxamate treatment whereas tumor suppressive regulators, including APC complex and RB, showed enhanced activities (Figure 3A, Figure S3A). In addition, literature-based analysis of upstream regulators for the eleven genes involved in microtubule regulation also indicated a highly significant association of E2Fs and RB in the regulation of these genes (Figure 3B, Figure S3B). Thus, these analyses strongly suggest the E2F-RB pathway as the most significant upstream candidate of lactate-driven cell motility regulation. We then experimentally examined the regulation of E2F1 in a lactate-enriched environment. First, we determined whether lactate-enriched environment regulates the transcriptional activity of E2F1 using a luciferase reporter assay system containing synthetic E2F1 binding motifs. E2F1-dependent luciferase value was significantly increased in metformin-treated HepG2 cells (Figure 3C). Exogenously provided lactate enhanced the E2F1 activity in Huh1 cells (Figure 3D), while inhibition of lactate production by oxamate treatment in HepG2/metR cells significantly decreased E2F1 activity (Figure 3E). We then investigated the mechanism of E2F activity regulation by lactate, and found that the protein expression of E2F1 was up-regulated by conditions leading to a high-level lactate accumulation, while down-regulated by the suppression of lactate production (Figure 3F,G,H). However, its mRNA levels were not changed in any of these conditions (Figure 3G,H), suggesting that lactate regulates E2F1 at the post-transcription level.

### 2.4. E2F1 Regulates Microtubule Dynamics to Promote Lactate-Dependent Cell Motility

**Figure 4:**
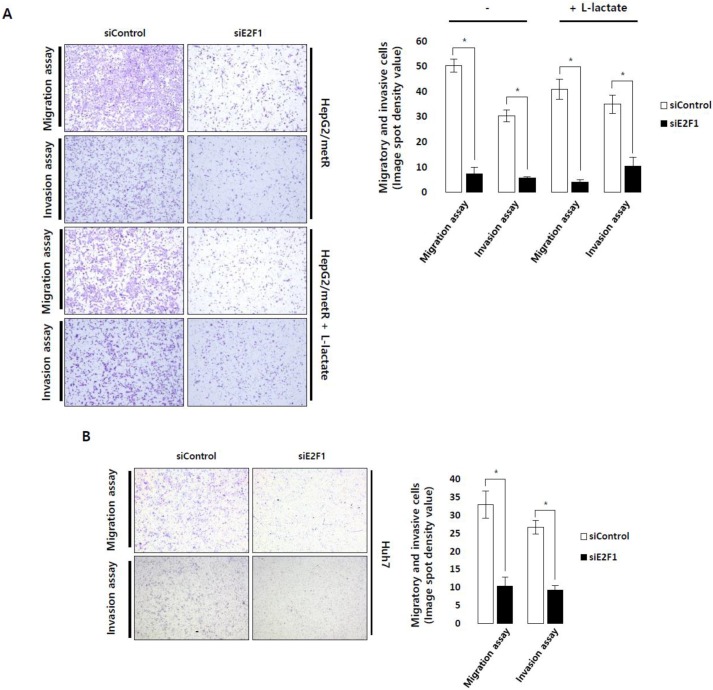

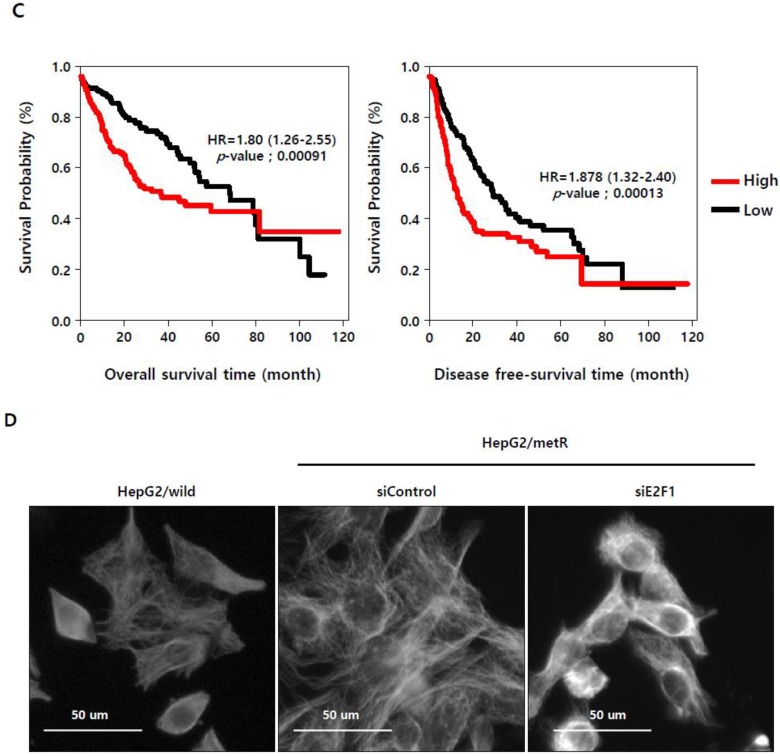
Role of E2F1 pathway in cell motility regulation. (**A**) Migration and invasion assays of E2F1-depleted HepG2/metR cells with or without exogenous l-lactate (30 mM). Original magnification (40×). (**B**) Migration and invasion assays of E2F1-depleted Huh7 cells. Original magnification (40×). (**C**) Overall survival and diseases-free survival rates of hepatocellular carcinoma patients depending on E2F1 activity in TCGA-LIHC (372 patients) cohort. Hazard Ratios with 95% confidence interval are also shown. (**D**) Immunofluorescence imaging of α-tubulin (white) protein in E2F1-depleted HepG2/metR cells is compared with those in control HepG2/metR and parental HepG2 cells. Experiments were conducted in triplicates, and the data shown represent a typical experiment. All quantitative data were obtained from three independent experiments, and shown as mean ± SD. * *p* < 0.05 and ** *p* < 0.01 vs. control by Student’s *t*-test.

Functional studies of E2F proteins as transcription factors clearly defined their roles in cell cycle control [22]. Furthermore, E2F target genes involved in metastasis or angiogenesis were also reported in breast cancer and melanoma [23,24,25]. However, roles of E2Fs in tumor metastasis have not been reported yet. We, therefore, experimentally examined the role of E2F1 in the regulation of lactate-induced cell motility. E2F1-depleted HepG2/metR cells displayed much reduced migratory and invasive properties compared to control cells (Figure 4A and Figure S4A). 

This impairment in cell motility could not be rescued by lactate supplementation in the culture medium, suggesting that E2F1 may function at the downstream in the lactate-induced cell motility regulation (Figure 4A). We also verified the decrease in migration and invasion properties in E2F1-depleted Huh7 cells (Figure 4B and Figure S4C). However, E2F1 depletion barely changed the growth rate of HepG2/metR and Huh7 cells during the migration and invasion assays (Figure S4B,D), suggesting that the E2F1-mediated cell motility control in lactate-enriched environment is not directly related to its cell cycle control capability (Figure 1G). We examined whether the E2F1-induced tumor cell motility is clinically relevant through Kaplan-Meier analysis. Estimation of the prognostic value for E2F1 activity in HCC patients using TCGA-LIHC (372 patients) data set indicated that elevated E2F1 activity was highly correlated with poor overall survival (*p* = 0.00091) and disease-free survival (*p* = 0.00013) (Figure 4C). Also, univariate analysis indicated that higher E2F1 activity is significantly associated with advanced tumor stages (*p* < 0.01) (Table S2). These results indicate that E2F1 may play a significant role in the prognosis of HCC patients by regulating tumor metastatic processes as well as tumor growth.

To investigate the cellular mechanism of E2F1-mediated cell motility regulation, we examined tubulin morphology depending on the expression of E2F1 in HepG2/metR cells by immunofluorescence imaging of α-tubulin protein. In HepG2/metR cells, α-tubulin was stained as long, straight fibers that are regularly oriented and spread throughout the cytoplasm (Figure 4D). However, in E2F1-depleted cells, tubulin fibers appeared to be randomly oriented, rarely assuming a stretched conformation and largely forming entangled structures within cells of compact shapes. The disrupted α-tubulin staining pattern was also observed in parental HepG2 cells (Figure 4D). This change in microtubule structure due to the E2F1 expression change was also confirmed in Huh7 cells (Figure S4E). These results indicate that E2F activity is closely associated with the activity of microtubule polymerization or assembly process, and suggest that lactate-induced E2F1 activation might play important roles in the regulation of microtubule dynamics and functions.

### 2.5. KIF Family Members Regulate Microtubule Dynamics and Cell Motility in an E2F1-Dependent Manner in the Lactate-Enriched Microenvironment

**Figure 5:**
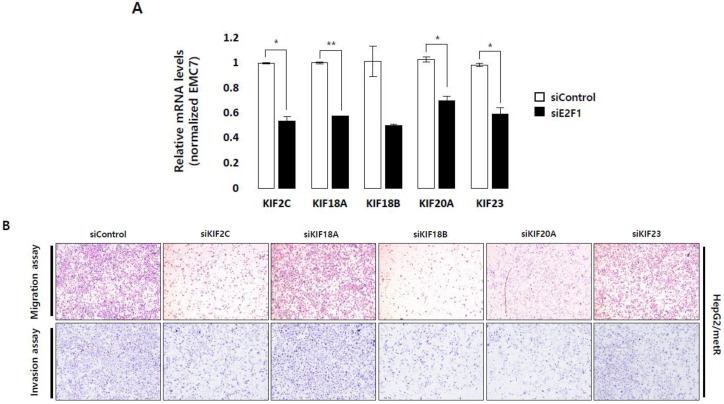

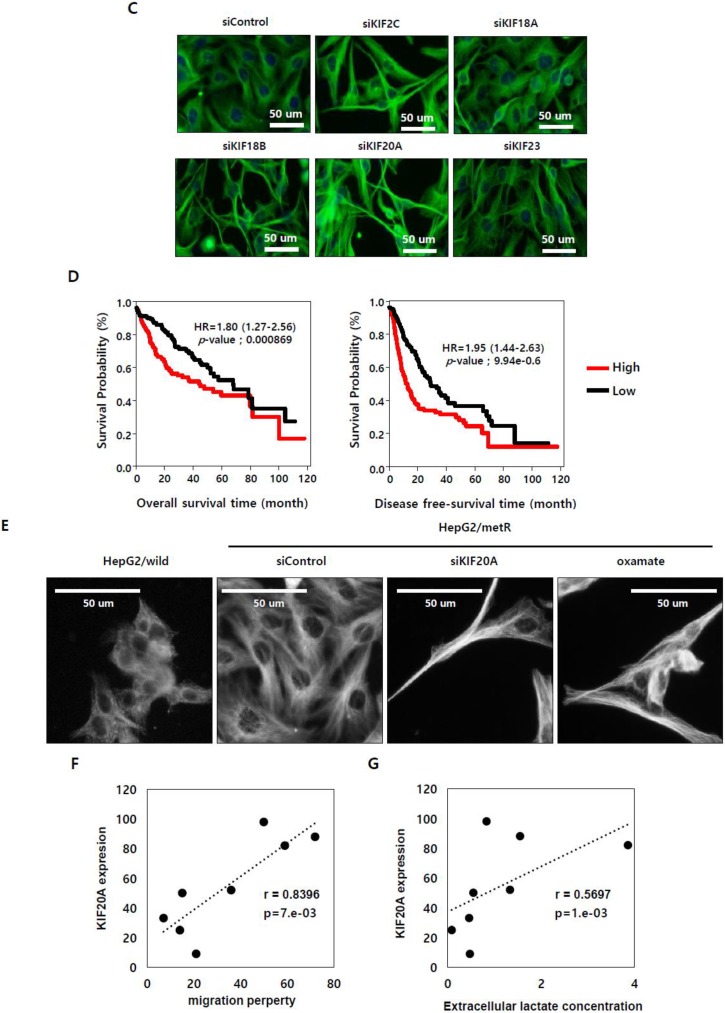

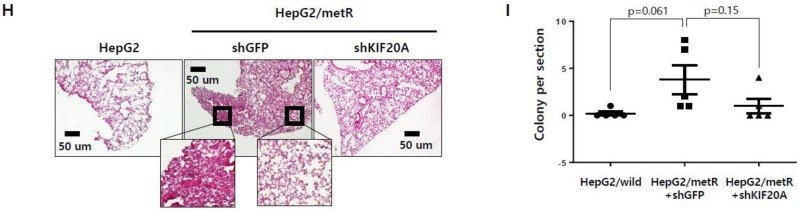
Lactate-induced cell motility is closely associated with E2F1-mediated activation of kinesin family genes and regulation of microtubule dynamics. (**A**) E2F1-dependence of the mRNA expression of 5 different microtubule-associated kinesin family genes was quantitatively evaluated in control and E2F1-depleted HepG2/metR cells. For qRT-PCR the expression level of transcripts for each gene was normalized by Ct-value of EMC7. Results from three independent experiments are shown as mean ± SD. The *p*-value was assessed by Student’s *t*-test. * *p* < 0.05 and ** *p* < 0.01 vs. control. (**B**) Migration and invasion assays to examine the effects of kinesin family gene depletion on the motility of HepG2/metR cells. Original magnification (40×). (**C**) Immunofluorescence imaging of α-tubulin protein (green) to examine the effects of kinesin family gene depletion on the tubulin fiber structure in HepG2/metR cells. Experiments were conducted in triplicate, and the data shown represent a typical experiment. (**D**) Overall survival and disease-free survival rates of hepatocellular carcinoma patients depending on the combined expression of KIF2C, KIF18B, and KIF20A in TCGA-LIHC (372 patients) cohort. Hazard Ratios with 95% confidence interval are also shown. (**E**) Immunofluorescence imaging of α-tubulin protein (white) in HepG2/wild cells, and control siRNA-treated, KIF20A-depleted, or oxamate-treated HepG2/metR cells. Experiments were conducted in triplicates, and the data shown represent a typical experiment. (**F**) Correlation analysis between KIF20A expression levels and migration properties in eight different liver cancer cell lines. (**G**) Correlation analysis between KIF20A expression levels and lactate production rates in eight different liver cancer cell lines. The r- and *p*-values were assessed by Spearman correlation calculation. (**H**) H&E stained sections of lungs isolated from mice that received tail vein injection of HepG2/wild, HepG2/metR+shGFP, and HepG2/metR+shKIF20A cells (scale bar; 50 µm). (**I**) Number of lung micrometastasis colonies per section in individual mice. Each data point represents the result for different mouse (*n* = 5 mice per group). Imaging of H&E-stained tissues, and microscopic counting of colonies were done using AlphaEaseFC program (area size 225 µm^2^). Error bars represent standard deviation; statistical analysis was performed using ANOVA test.

Given that lactate regulates cell motility by transcriptionally activating the genes functioning in cell-cell and cell-matrix communications, and that both the activity of E2F transcription factors and the mRNA expression of the five microtubule-associated kinesins are regulated in a lactate-dependent manner, we finally examined the role of E2F1 in lactate-induced cell motility regulation in relation to its ability to control the expression of kinesin genes. The mRNA levels of five kinesin family members were significantly reduced upon E2F1 depletion in HepG2/metR cells (Figure 5A). Motility assays indicated that depletion of KIF2C, KIF18B, and KIF20A in HepG2/metR cells significantly impaired their migratory and invasive properties whereas depletion of KIF18A and KIF23 did not (Figure 5B and Figure S5A). Furthermore, immunofluorescence staining of α-tubulin in HepG2/metR cells showed that depletion of KIF2C, KIF18B, and KIF20A causes severe agglomeration of tubulin fibers, while depletion of KIF18A and KIF23 does not (Figure 5C). These observations seem to be consistent with the migratory and invasive properties differentially affected by each kinesin gene depletion, suggesting that different kinesins may exert distinct roles in the lactate-induced regulation of microtubule functions. It is notable that depletion of these kinesins had no effects on the expression of different tubulins (Figure S5B). Kaplan-Meier analysis for the mRNA levels of KIF2C, KIF18B, and KIF20A using TCGA-LIHC (372 patients) data set indicated that combination of these three kinesins is associated with significantly poor prognosis in HCC patients with shorter overall survival (*p* = 0.000869) and disease-free survival (*p* = 9.94e−0.6) (Figure 5D). In addition, univariate analysis indicated that higher expression of the three kinesins is significantly associated with advanced tumor stages (*p* < 0.01) (Table S3).

We characterized the role of KIF20A in lactate-induced cell motility control in more detail. Immunofluorescence staining of α-tubulin in HepG2/metR cells showed that KIF20A depletion causes dramatic shrinkage and entangling of tubulin fibers compared to the well stretched structure observed in control cells (Figure 5E). The deformed tubulin structure was similar to what was observed in oxamate-treated HepG2/metR cells. We then examined the relationship between KIF20A expression level and migratory capacity of a cell using eight different liver cancer cell lines. A significantly high positive correlationship was observed between the KIF20A expression level and cellular migration capacity (Figure 5F and Figure S5D,E). Furthermore, KIF20A expression levels were closely associated with lactate production (Figure 5G and Figure S5D,F). Next, we examined the role of KIF20A in tumor metastasis in vivo by injecting HepG2/wild, HepG2/metR+shGFP, and HepG2/ metR+shKIF20A cells into the tail vein of BALB/c-nude mice (*n* = 5 per group). The intensity of nuclear RFP expression associated with metastasizing tumor cells, examined after 2 weeks of injection, was strongest in mice injected with HepG2/metR+shGFP cells, while it appeared much diminished in mice injected with KIF20A-depleted cells (Figure S5G). After 5 weeks, examination of lung micrometastasis using H&E stained histologic sections of lungs revealed that HepG2/metR+shGFP cells generated average 3.8 colonies/section in the lung metastasis whereas KIF20A-depleted cells generated average one colony/section (Figure 5H,I). None of the mice injected with HepG2/wild cells developed lung metastasis. These observations, together with the lactate dependence of KIF20A expression as shown in Figure 2, suggest that KIF20A plays an important role in microtubule dynamics and cell motility regulation, especially in the context of lactate-enriched environment of hepatocellular carcinomas, providing a potential therapeutic target for metastatic hepatocellular carcinoma.

## 3. Discussion

Lactate production is a dominant feature of malignant tumors. Generally, lactate accumulation in tumors is caused as the consequence of defects in cellular respiration, oncogenic alterations, and overexpression of glycolytic enzymes and metabolite transporters [26], and has been widely considered a key player of tumor progression [27]. Especially, elevated lactate is positively correlated with tumor metastasis, which is one of the hallmarks of malignancy [4]. On an experimental basis, exogenous lactate led to a concentration-dependent migration increment in various cell lines [28]. Some papers are suggesting the roles of lactate in tumor cell motility. Lactate enhances migratory capacity of gliomas by TGF-beta2-dependent regulation of matrix metalloproteinase-2 [10]. In addition, when exogenous lactate was added to cultured fibroblasts, increased hyaluronan promoted the motility and growth of tumor cells [29,30]. These data indicate that elevated lactate plays an important role in many aspects of invasion and metastasis of tumor cells. However, the molecular networks of key regulators directly associated with lactate-induced cell motility are not understood yet.

In this study, to investigate a mechanistic link between lactate accumulation and tumor cell motility, we identified lactate-responsive genes from the transcriptomic analysis of a metformin-induced hyper-glycolytic cell model. Literature-based bioinformatic analysis of top-ranked lactate targets indicated that genes regulating microtubule functions or dynamics are the most significantly enriched among others, suggesting that modulation of microtubules might be a major mechanism of lactate-induced cell motility regulation. Microtubules are polymeric structures that are modulated to orchestrate cellular movement, cell division, and intracellular transport when they control the cytoskeletal rearrangements via an intricate cross-talking network that involves interaction with diverse proteins and signaling molecules [31]. It has also been reported that the regulatory partners of microtubule dynamics are closely associated with tumor cell migration, invasion, and metastasis [31]. Among the lactate-responsive genes identified in this study are a group of kinesin family members, which are generally reported to affect microtubule dynamics including spindle assembly and kinetochore-microtubule attachment. Recent studies have highlighted the involvement of kinesins in the signaling pathways regulating cell migration. In lung adenocarcinoma, high KIF2A expression could regulate metastasis by affecting the PI3K/AKT and MAPK/ERK signaling pathways [18]. Furthermore, kinesin family 3A (KIF3A) over-expression in prostate cancer cells induced activation of Wnt/β-catenin pathway, leading to an acceleration of cell migration as well as proliferation of these cells [32]. APC could regulate cell migration by interacting with the microtubule plus-end-directed motor proteins, KIF3A-KIF3B [33]. In vascular endothelial cells, in response to vascular endothelial growth factor (VEGF) stimulation, KIF13B transports vesicles containing newly synthesized VEGF receptor 2 (VEGFR2) from Golgi to plasma membrane [34], and is required for VEGF-induced endothelial cell migration. KIF18A also promotes invasion and metastasis of hepatoma cells via MMP-7/MMP-9-related pathway [35]. High expression of KIF20A promotes tumor metastasis in various types of cancers [36,37,38]. These results have shown that elevated kinesins can regulate downstream signaling required for promoting tumor cell motility. In this study we showed that among the 5 kinesin members whose transcription is up-regulated in a lactate-dependent manner, depletion of KIF2C, KIF18B and KIF20A results in deregulation of microtubule structure as well as impairment of cell motility, providing experimental supports for the notion that lactate promotes cell motility by regulating microtubule functions through the induction of microtubule modulating genes such as kinesins.

Our sub-network enrichment analysis revealed a dominance of E2F-RB pathway among the putative upstream regulators of lactate-dependent gene expression. In addition, literature-based analysis on the eleven lactate-regulated genes involved in microtubule functions also indicated E2Fs as the highly significant upstream regulators. E2F1 is generally known as a transcription factor regulating cell cycle and proliferation. However, recent reports indicated a possibility that E2Fs might also have roles in cell motility regulation. Thus, E2F target genes were shown to regulate tumor metastasis in breast cancer and nasopharyngeal cancer [25,39]. In addition, E2F2 pathway was associated with relapse-free survival time of breast cancer patients [40]. We experimentally demonstrated that lactate activates E2F1-mediated gene transcription by inducing E2F1 protein accumulation in liver cancer cells, and that E2F1 depletion impairs lactate-induced cell motility concomitantly with the deregulation of tubulin structures. In addition, regarding the microtubule-associated kinesins whose expression is up-regulated by lactate, we observed that KIF20A expression is down-regulated at both protein and transcript levels upon E2F1 depletion in HepG2/metR cells (Figure S5C). We also showed that KIF2C and KIF18B, too, have a potential of mediating lactate-dependent cell motility, and that their expression is positively regulated by E2F1. KIF2C and KIF20A were reported, in a study based on ChIP-chip assays, as the target of E2F family including E2F1, E2F4, and E2F6 in normal and cancer cells of breast tissue [41]. Thus, our study strongly support the emerging roles of E2F transcription factors in cell motility regulation and tumor metastatic process, in addition to their well-characterized roles in cell cycle control, and exemplifies the E2F1-driven kinesin family gene expression as a crucial mechanism implicated in this process.

In summary, transcriptome analysis of a hyper-glycolytic cell model exhibiting highly enhanced motility suggested that the E2F pathway-mediated microtubule regulation might be the most significant mechanism of lactate-induced cell motility control. We then experimentally demonstrated that lactate activates E2F1-mediated gene expression whereby genes modulating microtubule functions, including a group of kinesin family members, are transcriptionally up-regulated. We also observed that E2F1 and its target kinesins such as KIF20A are required for modulating microtubules in a competent conformation to promote cell motility. These results provide a novel mechanistic link between lactate-induced cell motility and microtubule dynamics as well as an insight into how lactate-enriched tumor microenvironment promotes metastatic cancer progression.

## 4. Materials and Methods

### 4.1. Cell Culture

Human hepatocellular carcinoma cell lines HepG2, Huh1, and Huh7 were purchased from the American Type Culture Collection (ATCC, Manassas, VA, USA) and Japanese Collection of Research Bioresources Cell Bank (JCRB, Osaka, Japan). Cells were cultured in Dulbecco’s modified Eagle’s medium (LM001-65, Welgene, Gyeongsan, Republic of Korea) supplemented with 10% fetal bovine serum (16000-044, Gibco, Grand Island, NY, USA), 100 units/mL penicillin, and 100 µg/mL streptomycin (15140-122, Gibco) in a humidified atmosphere containing 5% CO_2_ at 37 °C.

### 4.2. Reagent Treatment

Cells were treated with 10 mM metformin (S1950, Selleckchem, Houston, TX, USA) by adding 1M stock solution to fresh medium. Sodium oxamate (O2751; LDHA inhibitor), l-lactate (L7022), α-cyano-4-hydroxycinnamic acid (C2020; MCT4 inhibitor), and rotenone (R8875) were purchased from Sigma (St. Louis, MO, USA), respectively.

### 4.3. Oxygen Consumption Rates and Extracellular Acidification Rates Measurement

To measure the mitochondrial respiratory activity in real time, the oxygen consumption rate (OCR) was monitored using the Seahorse XFp analyzer (Agilent, Santa Clara, CA, USA). Cells were plated at 90% confluency in a XFp 96-well plate in complete medium. The assay medium was Seahorse XF Base Medium (103335-100 and 103336-100, Agilent) supplemented with 25 mM glucose (Sigma, G8276), 10 mM pyruvate (P4562, Sigma), and 2 mM glutamine (21051-024, Gibco), pH 7.4. Assay cycles included 3 min of mixing and a 2-min waiting period, followed by 3 min of measurement. Following the measurement of basal respiration, oligomycin (1 µM) (75351, Sigma) was added, followed by FCCP (0.5 µM) (C2920, Sigma), and finally by rotenone and antimycin mix (1 µM) (R8875 and A8674, Sigma). The cells were then collected for BCA assay to normalize for the protein content (23228, Pierce, Waltham, MA, USA). Extracellular acidification rates (ECAR) were also monitored using the Seahorse XFp analyzer and proceeded in the same assay medium used in OCR measurement except for glucose. For the ECAR assay glucose (10 mM) was injected first into each well, followed by oligomycin (1 µM), and 2-DG (50 mM) (D6134, Sigma).

### 4.4. Extra- and Intracellular Lactate Measurement

Extracellular l-lactate concentration was measured using the EnzyChrom l-lactate Assay Kit (ECLC-100, BioAssay Systems, Hayward, CA, USA) according to the manufacturer’s protocol. Intracellular lactate levels were quantified by Lactate Assay Kit (MET-5012, Cell Biolabs, San Diego, CA, USA). During lactate level measurement, dialyzed FBS (26400-044, Gibco) was used.

### 4.5. Invasion and Migration Assay

Cell migration and invasion assays were performed using Transwell chamber (8 µm pore size) (353097, Falcon, Corning, NY, USA). Cells (2 × 10^4^) were resuspended in 200 µL of serum-free growth medium for both cell migration and invasion assays. 10% (*v*/*v*) calf serum was added to the lower chamber as the chemoattractant. After incubation for 24 h at 37 °C, cells on upper surface were removed and cells migrated to the lower surface were fixed and stained for 20 min with 100% cold methanol, and 0.25% crystal violet (C3886, Sigma). For invasion assay, the cells were added to the interior of the inserts pre-coated with 10 mg/mL of growth factor-reduced Matrigel (354234, Corning Incorporated, Corning, NY, USA), and then processed as described above for the migration assay.

### 4.6. RNA-Sequencing for Expression Profiling

To measure the gene expression changes induced by lactate-enriched microenvironment, total RNA from parental HepG2 cells, HepG2/metR cells, and oxamate-treated HepG2/metR cells were analyzed using the NextSeq^®^ 500 (Illumina, San Diego, CA, USA). RNA was extracted using Trizol (15596-018, Invitrogen, Carlsbad, CA, USA). We identified all the genes demonstrating < 2-fold increase in HepG2/metR vs. parental HepG2 but < 1.5-fold decrease by oxamate treatment of HepG2/metR cells. The GEO accession number for the RNA-seq data is GSE125525. Using real-time RT-PCR, the RNA-seq data for select genes were validated across the samples. Filtered and normalized RNA-seq data were further analyzed using the Pathway Studio software to estimate the regulatory activity (Z-score) of putative upstream regulators, or to identify target pathways commonly modulated by a group of differentially expressed genes or their upstream regulators based on literature mining. For detailed procedures one might refer to Pathway Studio (Version 11.1).

### 4.7. Transfection of Small-Interfering RNAs

Cells were plated at 50–60% confluency on day 0 in the complete medium. On day 1, the cells were transfected with siRNAs against MCT4 (siMCT4), KIF2C (siKIF2C), KIF18A (siKIF18A), KIF18B (siKIF18B), KIF20A (siKIF20A), KIF23 (siKIF23), E2F1 (siE2F1), and negative control (siControl) at a concentration of 40 nM using Lipofectamine RNAiMAX reagent (13778-150, Invitrogen) according to the manufacturer’s protocol. For each gene the siRNA consisted of a mixture of 2–4 different sequences, and purchased from Dharmacon (On-Targetplus SMARTpool, Lafayette, CO, USA) or Bioneer (AccuTarget ™, Daejeon, Republic of Korea). Assays were performed 48 h after transfection.

### 4.8. Quantitative Real-Time RT-PCR

Total RNA was isolated from cultured cells using Trizol according to the manufacturer’s protocol. For quantitative real-time RT-PCR (qRT-PCR) analysis of mRNA, 500 ng of total RNA was transcribed with oligo-dT and reverse transcriptase (EP0442, Thermo, Waltham, MA, USA). Quantitative PCR was then performed using a SYBR Green PCR Master Mix (1708882, Bio-Rad, Hercules, CA, USA) on a CFX analyzer (Bio-rad). The primers used in qRT-PCR are shown in Supplementary Table S4. ACTB and EMC7 were used as the endogenous control for normalization. Each reaction was performed in triplicates and repeated in at least three different samples.

### 4.9. Reporter Vector Constructs and Luciferase Reporter Assay

pGL4 vector (E6661, Promega, Madison, WI, USA) containing luciferase gene under the control of putative E2F1 binding sequences was constructed. 293T cells were plated in 24-well plates at 3 × 10^4^ cells/well and grown to 60% confluence. Cells were transfected with mixtures containing 100 ng of the pGL4 reporter construct carrying E2F1 binding sequences and *renilla* control vector using Lipofectamin 3000 (L3000-015, Invitrogen) as described in the manufacturer’s protocol. After 24 h of transfection, the medium was changed with the one containing lactate, metformin, or oxamate. Dual luciferase reporter assay system (E1910, Promega) was used with multiple microplate reader (BioTek, Winooski, VT, USA).

### 4.10. Western Blot Analysis

Cells were collected and lysed in RIPA buffer containing Halt™ Protease and Phosphatase Inhibitor Cocktail (78441, Thermo Scientific). After incubating for 30 min on ice with vortexing every 10 min, cellular debris was removed by centrifugation at 13,000 rpm for 30 min at 4 °C. Proteins were separated on an 8–12% SDS-polyacrylamide gel. After electrophoresis, protein was transferred to a nitrocellulose membrane (66485, Pall, Port Washington, NY, USA) and incubated with appropriate primary antibodies at 4 °C overnight. The following primary antibodies were used: E2F1 rabbit polyclonal antibody (SC-193, Santa Cruz, Santa Cruz, CA, USA), KIF20A mouse monoclonal antibody (SC-374508, Santa Cruz), β-actin mouse monoclonal antibody (SC-47778, Santa Cruz), α-tubulin mouse monoclonal antibody (SC-23948, Santa Cruz), β-tubulin mouse monoclonal antibody (SC-5274, Santa Cruz), and γ-tubulin rabbit polyclonal antibody (GTX13286, Gene Tex, Irvine, CA, USA). Beta-actin was used as the loading control. Membranes were incubated with corresponding horse radish peroxidase-conjugated secondary antibodies for 1h at room temperature. The following secondary antibodies were used: HRP-linked anti-mouse IgG antibody (AP124P, Millipore, Burlington, MA, USA), and HRP-linked anti-rabbit IgG antibody (AP132P, Millipore). The blotted proteins were visualized by using Western ECL solution (34080, Pierce) and images were obtained by using chemiluminescent image analyzer (LAS-4000, Fujifilm, Tokyo, Japan).

### 4.11. Survival Analysis

Liver cancer open database, TCGA-LIHC cohort RNA-seqV2 data including 372 tumors patients were downloaded from TCGA data portal (http://portal.gdc.cancer.gov/). Time-to-event (“survival”) analysis was performed using Cox proportional hazards models. Statistical significance was assessed using the log-rank (score) test. Kaplan-Meier curves were also constructed in order to plot the percentage probability of patients’ DFS (disease free-survival) and OS (overall survival), after their classification into low (< median) or high (≥ median) of Z-scores of the E2F1-Q3 target gene set (244 genes from GSEA of the Broad Institute (http://software.broadinstitute.org/gsea/msigdb)) or the mRNA expression of kinesin family genes (KIF2C, KIF18B and KIF20A). All computation was performed using the survival package (Version 2.43-3) in the R statistical programming environment (http://cran.r-project.org/) (Version 3.4.3).

### 4.12. Immunofluorescence

To visualize microtubules, cells were plated on a chamber slide (154526, Nunc, Waltham, MA, USA) in complete medium. Cells were fixed with 100% cold methanol for 10 min at room temperature. Immunostaining was carried out using a mouse anti-α-tubulin antibody and detected by an Alexa Fluor 488-, or 594-conjugated secondary antibody (A11029 and A11032, Invitrogen). DAPI (1 mg/mL) was used for fluorescent staining of nuclear DNA (62248, Invitrogen). Fluorescence images were acquired on a Nikon IntensLight microscrope (Nikon, Tokyo, Japan).

### 4.13. Tail-Vein Injection Model

HepG2/wild, HepG2/metR+shGFP and HepG2/metR+shKIF20A cells (each 10^6^ cells) were resuspended in PBS and injected into the tail vein of male BALB/c-nude mice (aged 5 weeks; 18–23 g). Prior to the injection cells were pre-incubated overnight in a complete medium containing CellLight^®^ Nucleus-RFP (C10603, Molecular Probes, Eugene, OR, USA) to help track them in the subsequent image analysis. After 14 days, regions of a live mouse body were imaged to define RFP signals using IVIS Luminal system and Ivis live imaging software (PerkinElmer, Waltham, MA, USA). Contents dealing with animal subjects were approved by the Institutional Animal Care and Use Committee of Korea Research Institute of Bioscience and Biotechnology (KRIBB) (ethical code: KRIBB-AEC-18091, permission date: 2 April 2018).

### 4.14. Hematoxylin and Eosin Staining

Mouse lungs were resected out and fixed with 3.7% paraformaldehyde for the evaluation of metastasizing tumors. Tissues embedded in OCT compound (4583, Tissue-Tek, Torrance, CA, USA) were sectioned (9 μm), mounted, and then stained for 13 min with hematoxylin (HEM001, Biopure, Chungju, Republic of Korea) followed by eosin (HT110132, Sigma) for 5 min.

## 5. Conclusions

In this study we showed that lactate activates E2F pathways through a posttranscriptional mechanism to up-regulate the expression of genes functioning in cell-cell or cell-matrix communications. In particular, genes modulating microtubule functions were up-regulated in a lactate-dependent manner, and, among others, the pro-metastatic roles of a group of kinesin family genes (KIF2C, KIF18B, KIF20A) were experimentally demonstrated in the context of lactate- and E2F1-dependent regulation of microtubule functions and cell motility. This study provides a novel mechanistic link between lactate-induced cell motility and microtubule dynamics as well as insights into how tumor-associated lactate promotes metastatic progression of malignant cancers.

## Supplementary Materials

The following are available online at https://www.mdpi.com/2072-6694/11/3/274/s1, Figure S1: Lactate-enriched microenvironment affects migratory and invasive properties in hepatocellular carcinoma cells; Figure S2: Lactate-dependent expression of kinesin family genes; Figure S3: Identification of E2F-RB pathway as the dominant upstream regulator of lactate-responsive gene expression; Figure S4: siRNA-mediated knockdown of E2F1 expression and its effects on microtubule dynamics; Figure S5: Roles of kinesin family genes in the regulation of microtubule dynamics and lactate-induced cell motility; Table S1: List of the genes directly responding to lactate signaling; Table S2: Univariate analysis of variables associated with E2F1 activity in TCGA LIHC cohort; Table S3: Univariate analysis of variables associated with KIF gene set (KIF2C, KIF18B, and KIF20A) activity in TCGA LIHC cohort; Table S4: List of the primers’ sequences.
