# Supplementary material for: Lactate Activates the E2F Pathway to Promote Cell Motility by Up-Regulating Microtubule Modulating Genes
Source: Cancers (Basel). 2019 Feb 26;11(3):274. doi: 10.3390/cancers11030274 (PMC6468617; doi:10.3390/cancers11030274)
Supplement: Supplementary file 1 [file cancers-11-00274-s001.pdf]

cancers

Supplementary materials: Lactate Activates E2F Pathway to Promote Cell Motility by Up-Regulating Microtubule Modulating Genes

Yi-deun Jung, Jung Hee Cho, Seulki Park, Minho Kang, Seung-jin Park, Dong Hee Choi, Moonkyung Jeong, Kyung Chan Park, Young Il Yeom and Dong Chul Lee

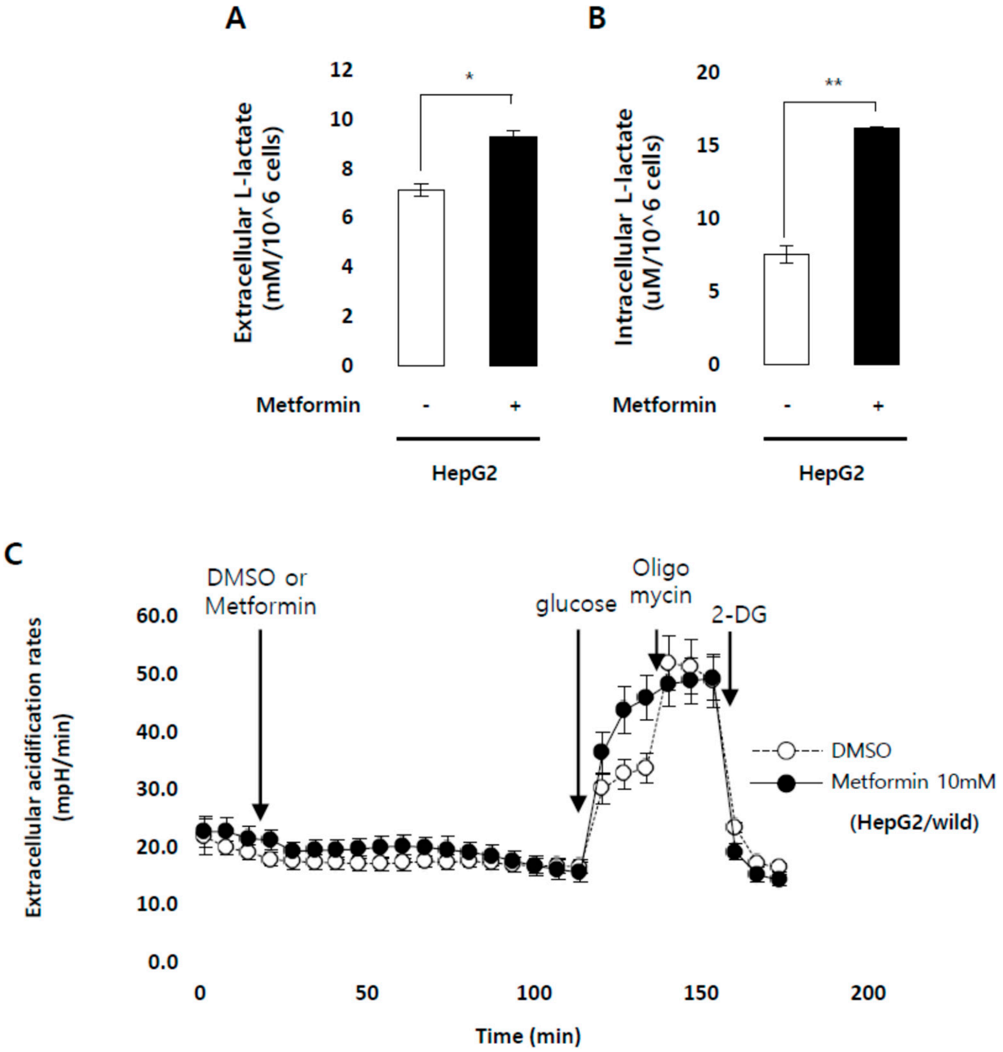

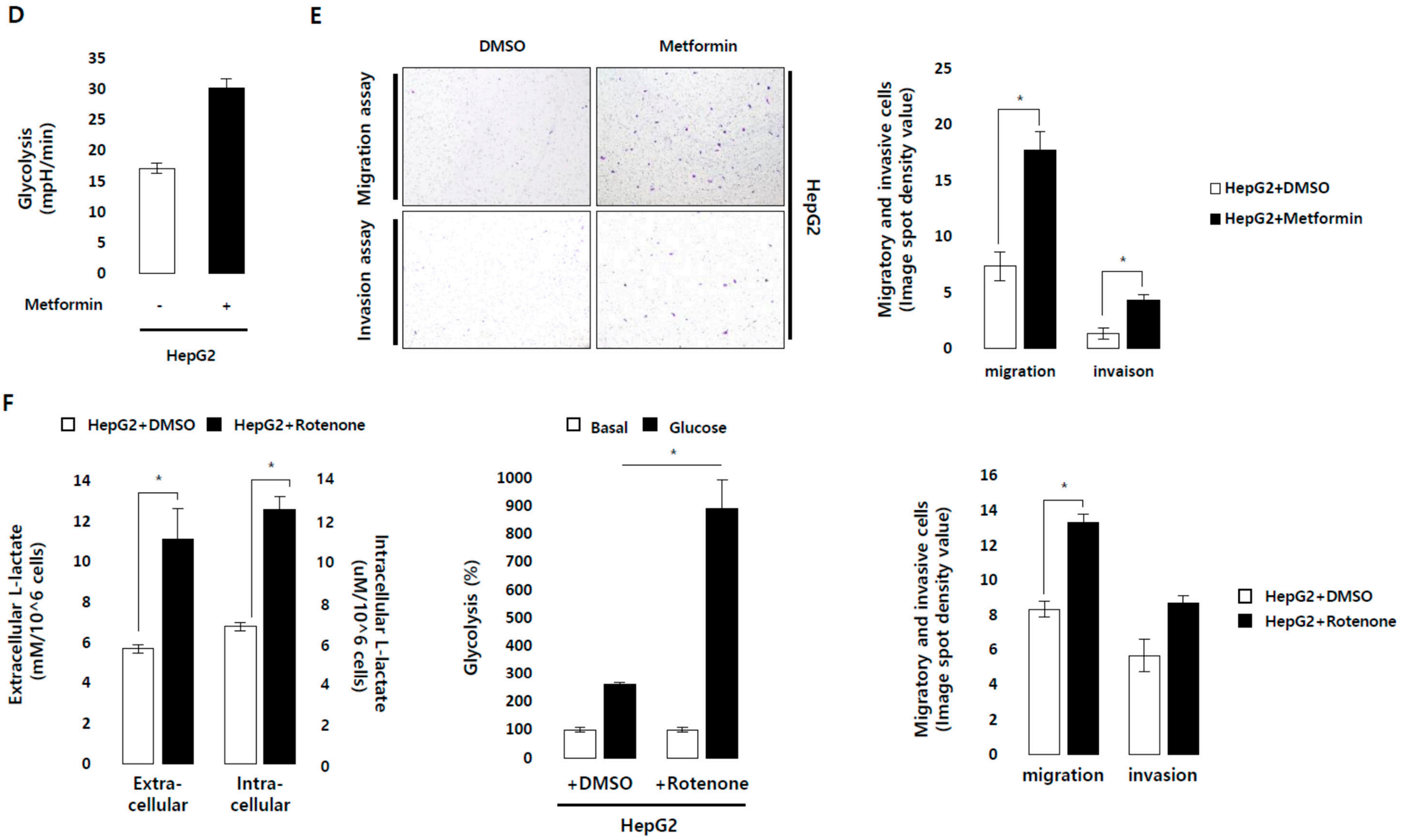
D
Glycolysis (mpH/min)
35
30
25
20
15
10
5
0
Metformin
-
+
HepG2
E
DMSO
Metformin
Migration assay
Invasion assay
HepG2
Migratory and invasive cells (Image spot density value)
25
20
15
10
5
0
migration
invaison
HepG2+DMSO
HepG2+Metformin
F
HepG2+DMSO
HepG2+Rotenone
Extracellular L-lactate (mM/10^6 cells)
Intracellular L-lactate (uM/10^6 cells)
14
12
10
8
6
4
2
0
Extra-cellular
Intra-cellular
Basal
Glucose
Glycolysis (%)
1000
900
800
700
600
500
400
300
200
100
0
+DMSO
+Rotenone
HepG2
Migratory and invasive cells (Image spot density value)
16
14
12
10
8
6
4
2
0
migration
invasion
HepG2+DMSO
HepG2+Rotenone

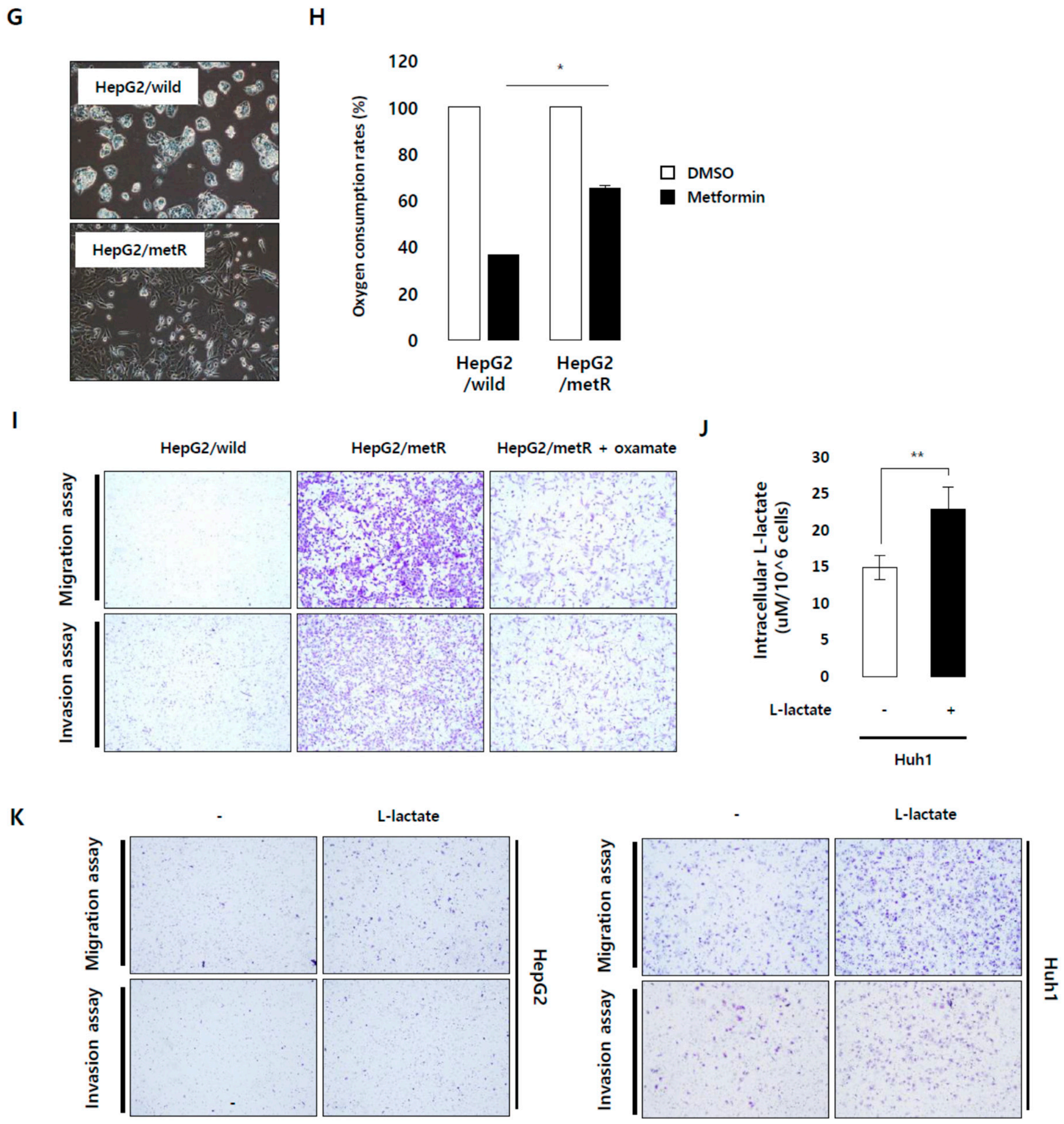

Figure S1. Lactate-enriched microenvironment affects migratory and invasive properties in hepatocellular carcinoma cells. (**A**,**B**). Extra- and intra-cellular lactate production by HepG2 cells after transient treatment with metformin (10 mM). (**C**) Kinetic ECAR response of HepG2 cells to metformin (10 mM) or DMSO, glucose (20 mM), oligomycin (1 uM), and 2-DG (50 mM), respectively. The ECAR values were normalized by the BCA protein assay. (**D**) Glycolysis rates measured after the addition of a saturating amount of glucose to DMSO- or metformin-treated HepG2 cells. (**E**) Changes in migratory and invasive properties induced in HepG2 cells after a transient treatment with metformin. Experiments were conducted in triplicates, and the image data shown represent a typical experiment. Results from three independent experiments are shown as mean ± SD. (**F**) Changes in lactate production, glycolysis rates, and migratory properties in HepG2 cells after a transient treatment with rotenone (2 uM). DMSO was used for the control. The ECAR values, which represents cellular glycolysis rates, were normalized by BCA protein assay, and presented as the percentage increase relative to the basal ECAR value for DMSO- or rotenone-treated condition. (**G**) Morphological changes induced in a hyper-glycolytic model of HepG2 cells that acquired a long-term resistance to metformin (HepG2/metR). They typically showed features of flat, large, and irregular shapes in contrast to parental HepG2 cells. (**H**) Oxygen consumption rates of HepG2 and HepG2/metR cells treated with metformin (10 mM). The OCR values were normalized by BCA protein assay. Metformin-induced change in OCR is presented as the percentage relative to the basal OCR value of each cell

type (DMSO). (**I**) Enhanced migration and invasion properties of HepG2/metR cells are abrogated by oxamate treatment. Data for parental HepG2 cells are shown for comparison. Experiments were conducted in triplicates, and the data shown represent a typical experiment. (**J**) Intracellular lactate level measured after a transient treatment of exogenous L-lactate (30 mM) in Huh1 cells. Results from three independent experiments are shown as mean ± SD. (**K**) Changes in migratory and invasive properties induced in HepG2 and Huh1 cells after a transient treatment with exogenous L-lactate. Experiments were conducted in triplicates, and the data shown represent a typical experiment. * $p < 0.05$ and ** $p < 0.01$ vs. control. Magnification for subfigure E,G,I,K: ×40.

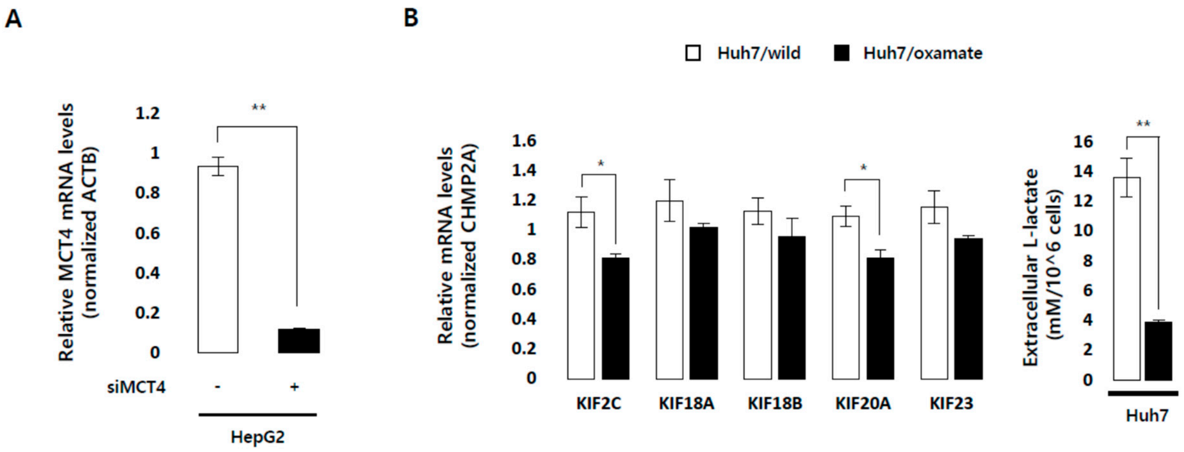

Figure S2. Lactate-dependent expression of kinesin family genes. (**A**) siRNA-mediated depletion of MCT4 gene expression in HepG2 cells. (**B**) Changes in mRNA expression levels of kinesin family genes were quantitatively measured in Huh7 cells after oxamate treatment. Changes in extracellular lactate concentration due to the oxamate treatment are shown on the right. Results from three independent experiments are shown as mean ± SD. For qRT-PCR the expression level of transcripts for each gene was normalized by Ct-value of CHMP2A. The *p*-value was assessed by Student's t-test. * $p < 0.05$ and ** $p < 0.01$ vs. control.

A

	Z-score	-log2(p-value)
E2F4	-0.816497	25.15090571
RB1	3.18198	19.77376166
E2F	-1.50756	18.37752498
APC/C	4.12311	16.94284208
E2F3	-1.5	16.00552169
E2F2	-1.50756	14.13685899
E2F1	-1.60451	14.09772467
CDKN1A	1.04257	13.6188072
eIF2	-0.447214	12.79265173
MNT	1	11.8782617

B

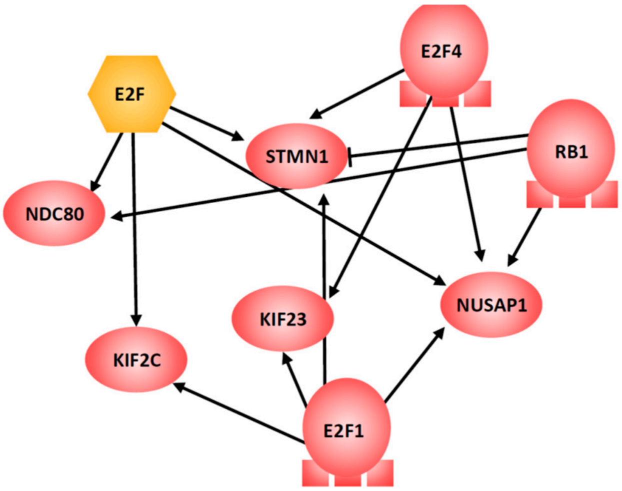

Figure S3. Identification of E2F-RB pathway as the dominant upstream regulator of lactate-responsive gene expression. (**A**) Profiling of upstream regulators of lactate-induced gene expression in oxamate-treated HepG2/metR cells through a sub-network enrichment analysis. The activation scores (Z-score) for putative upstream regulators and their statistical significance are shown. (**B**) Identification of E2F-RB pathway as the upstream regulator of the eleven lactate-responsive genes involved in microtubule regulation through the literature mining process of Pathway Studio.

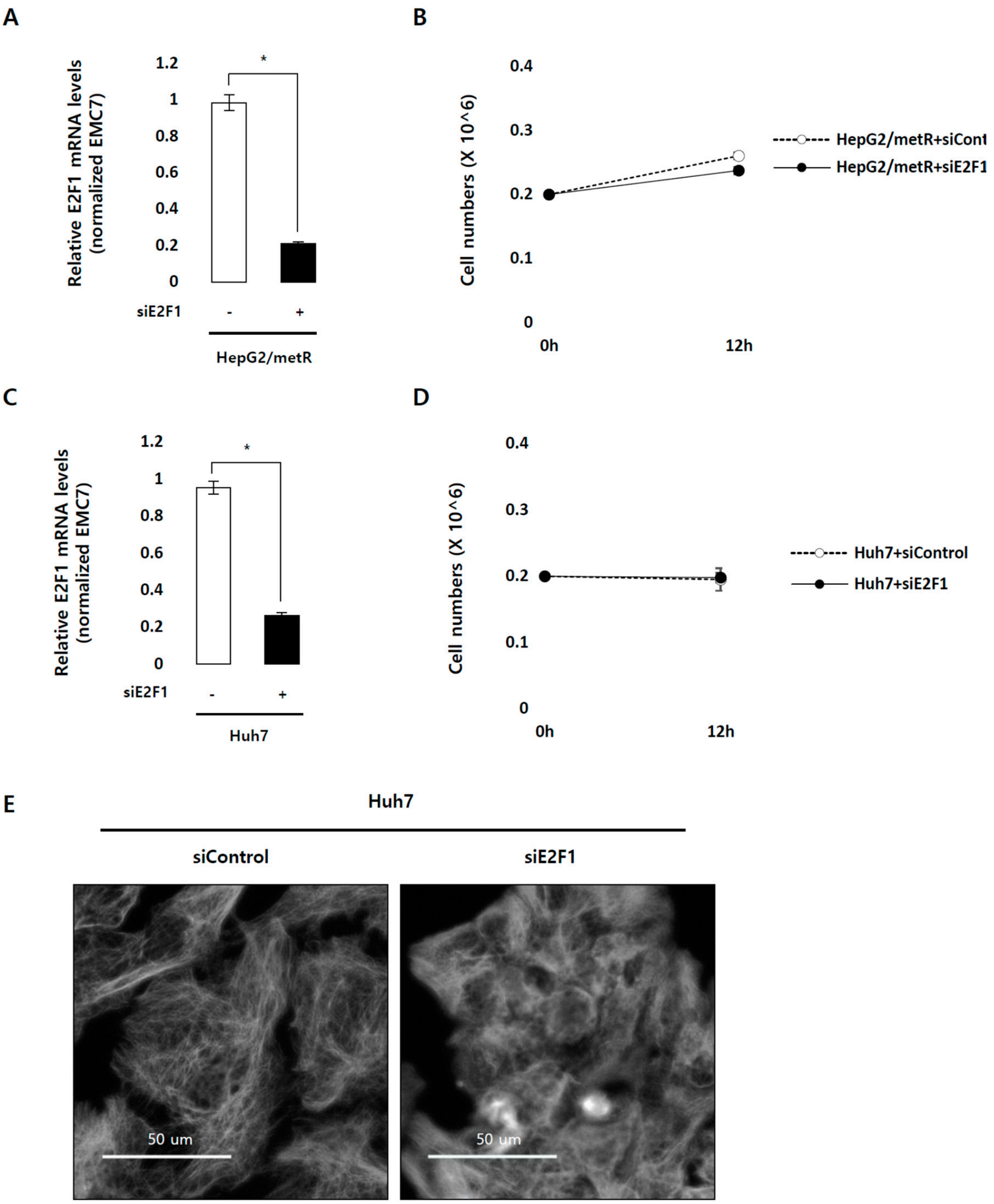

Figure S4. siRNA-mediated knockdown of E2F1 expression and its effects on microtubule dynamics. (**A**) siRNA-mediated knockdown of E2F1 expression in HepG2/metR cells. (**B**) Cell growth profiles of control or E2F1-depleted HepG2/metR cells during migration and invasion assays (12 h). (**C**) siRNA-mediated knockdown of E2F1 expression in Huh7 cells. (**D**) Cell growth profiles of control or E2F1-depleted Huh7 cells. (**A**,**C**) For qRT-PCR the expression level of transcripts for each gene was normalized by Ct-value of EMC7. Results from three independent experiments are shown as mean ± SD. The *p*-value was assessed by Student's t-test. (**E**) Immunofluorescence imaging of α-tubulin protein (white) in E2F1-depleted Huh7 cells. Experiments were conducted in triplicates, and the data shown represent a typical experiment. * $p < 0.05$ vs. control.

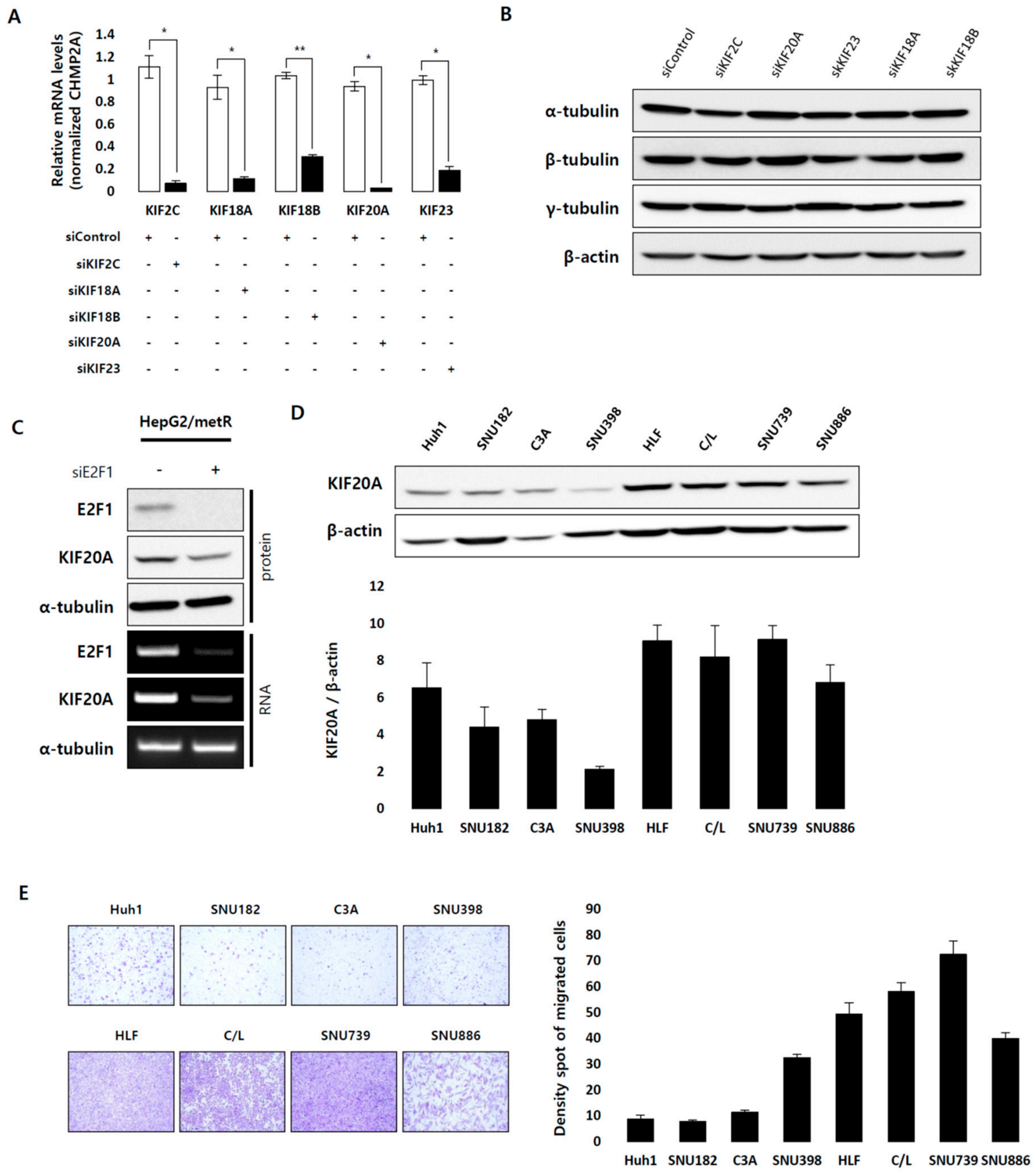
A
Relative mRNA levels (normalized CHMP2A)
1.4
1.2
1
0.8
0.6
0.4
0.2
0
*
**
KIF2C
KIF18A
KIF18B
KIF20A
KIF23
siControl
siKIF2C
siKIF18A
siKIF18B
siKIF20A
siKIF23
B
siControl
siKIF2C
siKIF20A
siKIF23
siKIF18A
siKIF18B
α-tubulin
β-tubulin
γ-tubulin
β-actin
C
HepG2/metR
siE2F1
E2F1
KIF20A
α-tubulin
protein
RNA
D
Huh1
SNU182
C3A
SNU398
HLF
C/L
SNU739
SNU886
KIF20A
β-actin
KIF20A / β-actin
12
10
8
6
4
2
0
E
Density spot of migrated cells
90
80
70
60
50
40
30
20
10
0

F

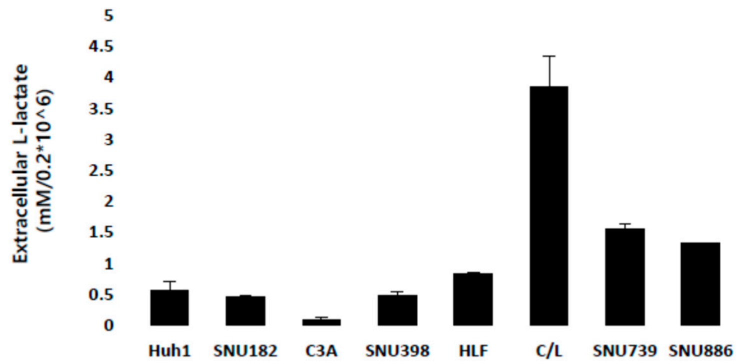

G

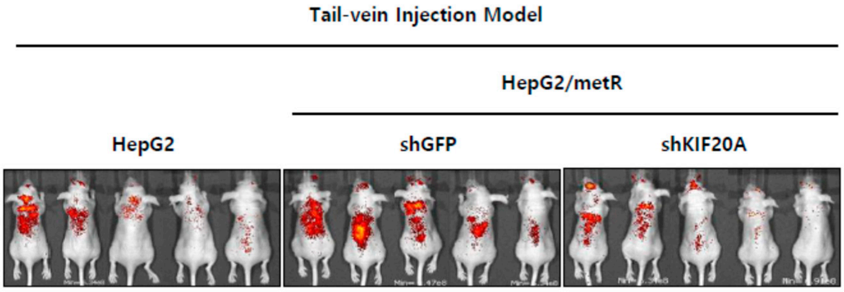

Figure S5. Roles of kinesin family genes in the regulation of microtubule dynamics and lactate-induced cell motility. (**A**) Verification of siRNA-mediated depletion of kinesin family gene expression in HepG2/metR cells by qRT-PCR. For qRT-PCR the expression level of transcripts for each kinesin gene was normalized by Ct-value of CHMP2A. Results from three independent experiments are shown as mean ± SD. The *p*-value was assessed by Student's t-test. (**B**) Examination of the effects of kinesin gene depletion on the expression of different tubulins in HepG2/metR cells. Protein expression of tubulin family genes was estimated by Western blotting. (**C**) Protein and mRNA expression of KIF20A in E2F1-depleted HepG2/metR cells. Experiments were conducted in triplicates, and the data shown represent a typical experiment. (**D**) KIF20A protein expression in eight different liver cancer cell lines was estimated by Western blotting followed by densitometry. (**E**) Estimation of the relative migration capacity of eight different liver cancer cell lines. Magnification: 40×. (**F**) Extracellular lactate levels measured in eight different liver cancer cell lines. Results from three independent experiments are shown as mean ± SD. (**G**) Tracking of metastasizing liver cancer cells in live mice using a live imaging software. Imaging was carried out after 2 weeks of cell injection into the tail vein of BALB/c-nude mice. * $p < 0.05$ and ** $p < 0.01$ vs. control.

Table S1. List of the genes directly responding to lactate signaling.

Gene Symbol	Ensembl ID (or Entrez Gene)	Gene Name	HepG2/wild-HepG2/metR Log_2 Fold Change	HepG2/metR-HepG2/metR+Oxamate Log_2 Fold Change
SRGN	ENSG00000122862	Serglycin	8.663534	−0.70503616
ANXA1	ENSG00000135046	Annexin A1	7.9717927	−0.7499008
G0S2	ENSG00000123689	G0/G1 Switch 2	7.180892	−0.68959665
PLP2	ENSG00000102007	Proteolipid Protein 2	6.461257	−0.5630083
KRTAP2-3	ENSG00000212724	Keratin Associated Protein 2-3	6.4068155	−1.234129
NRG1	ENSG00000157168	Neuregulin 1	5.9283357	−0.5623679
SLC43A3	ENSG00000134802	Solute Carrier Family 43 Member 3	5.9182715	−0.73265934
MYEOV	ENSG00000172927	Myeloma Overexpressed	5.56702	−0.7658987
MOK	ENSG00000080823	MOK Protein Kinase	5.477459	−0.52942085
SNHG15	ENSG00000232956	Small Nucleolar RNA Host Gene 15	5.134816	−1.0345192
FOXD1	ENSG00000251493	Forkhead Box D1	4.8395553	−0.541657
CD82	ENSG00000085117	CD82 Molecule	4.770322	−0.6132164
PLAT	ENSG00000104368	Plasminogen Activator, Tissue Type	4.633147	−0.50059414
CHAC1	ENSG00000128965	ChaC Glutathione Specific Gamma-Glutamylcyclotransferase 1	4.455889	−2.2658846
PLAU	ENSG00000122861	Plasminogen Activator, Urokinase	4.341663	−1.2562137
TGM2	ENSG00000198959	Transglutaminase 2	4.299946	−0.54446316
SPARC	ENSG00000113140	Secreted Protein Acidic And Cysteine Rich	4.2835507	−0.55296993
RPL21	ENSG00000122026	Ribosomal Protein L21	4.234487	−0.9239664
IFI27L2	ENSG00000119632	Interferon Alpha Inducible Protein 27 Like 2	4.1375957	−0.56406116
BIRC3	ENSG00000023445	Baculoviral IAPBaculoviral IAP Repeat Containing 3 Repeat Containing 3	4.019673	−0.5170846
FOSL1	ENSG00000175592	FOS Like 1, AP-1 Transcription Factor Subunit	3.947637	−0.7359171
SHISA2	ENSG00000180730	Shisa Family Member 2	3.853442	−0.5062511
INSIG1	ENSG00000186480	Insulin Induced Gene 1	3.8162234	−1.1338937
SOCS3	ENSG00000184557	Suppressor Of Cytokine Signaling 3	3.7340534	−0.50167465
RIMKLB	ENSG00000166532	Ribosomal Modification Protein RimK Like Family Member B	3.5299654	−0.5982802
PLAUR	ENSG00000011422	Plasminogen Activator, Urokinase Receptor	3.4646206	−0.51312256
TUBB6	ENSG00000176014	Tubulin Beta 6 Class V	3.4066448	−0.6411624
WDR54	ENSG00000005448	WD Repeat Domain 54	3.3614988	−0.9210162
UAP1	ENSG00000117143	UDP-N-Acetylglucosamine Pyrophosphorylase 1	3.1766644	−0.8585243
HAS3	ENSG00000103044	Hyaluronan Synthase 3	3.0806127	−0.5995364
ULBP1	ENSG00000111981	UL16 Binding Protein 1	3.0395777	−0.70952964
C15orf48	ENSG00000166920	Chromosome 15 Open Reading Frame 48	3.020333	−0.6276443
CLDN10	ENSG00000134873	Claudin 10	2.9294827	−0.591835
FLJ23867	Entrez Gene:200058	Uncharacterized Protein FLJ23867	2.8299212	−0.7818744
SNORA70	ENSG00000207165	Small Nucleolar RNA, H/ACA Box 70	2.81736	−1.902751

LINC01279	Entrez Gene:100506621	Long Intergenic Non-Protein Coding RNA 1279	2.7018592	−0.5433452
RRAS	ENSG00000126458	RAS Related	2.6430616	−0.52122164
KRT15	ENSG00000171346	Keratin 15	2.637089	−0.5660372
NRGN	ENSG00000154146	Neurogranin	2.6237876	−0.6923499
UBE2S	ENSG00000108106	Ubiquitin Conjugating Enzyme E2 S	2.619019	−0.5673156
FLJ45513	ENSG00000204584	Uncharacterized LOC729220	2.6142166	−0.6827209
CXCL2	ENSG00000081041	C-X-C Motif Chemokine Ligand 2	2.5624237	−1.4232312
LEPR	ENSG00000116678	Leptin Receptor	2.505877	−0.50919724
RDM1	ENSG00000278023	RAD52 Motif Containing 1	2.472673	−0.61161304
APOBEC3B	ENSG00000179750	Apolipoprotein B MRNA Editing Enzyme Catalytic Subunit 3B	2.461593	−0.7941332
ANGPTL4	ENSG00000167772	Angiopoietin Like 4	2.440999	−1.1044414
C8orf46	ENSG00000169085	Vexin	2.281881	−0.66461015
CARS	ENSG00000110619	Cysteinyl-TRNA Synthetase	2.247805	−0.66027355
LFNG	ENSG00000106003	LFNG O-Fucosylpeptide 3-Beta-N-Acetylglucosaminyltransferase	2.2101269	−0.6740203
WDR76	ENSG00000092470	WD Repeat Domain 76	2.177379	−0.61951613
CCDC74A	ENSG00000163040	Coiled-Coil Domain Containing 74A	2.1341832	−0.5484978
NBL1	ENSG00000158747	NBL1, DAN Family BMP Antagonist	1.9864647	−0.92279935
LGALS1	ENSG00000100097	Galectin 1	1.9608679	−0.66515255
KIF18A	ENSG00000121621	Kinesin Family Member 18A	1.9236305	−0.50712156
UCP2	ENSG00000175567	Uncoupling Protein 2	1.9134188	−1.3448291
MIR17HG	ENSG00000215417	MiR-17-92a-1 Cluster Host Gene	1.8753912	−0.514632
TMEM161B-AS1	ENSG00000247828	TMEM161B Antisense RNA 1	1.8367593	−1.198487
KIF23	ENSG00000137807	Kinesin Family Member 23	1.8086615	−0.64275074
CATSPER1	ENSG00000175294	Cation Channel Sperm Associated 1	1.7882096	−0.6271752
STMN1	ENSG00000117632	Stathmin 1	1.7763343	−0.6696081
FAM72B	ENSG00000188610	Family With Sequence Similarity 72 Member B	1.7376454	−0.6952479
CENPI	ENSG00000102384	Centromere Protein I	1.7014825	−0.53399706
SLMO2-ATP5E	Entrez Gene:100533975	SLMO2-ATP5E Readthrough	1.6976035	−0.630363
NUSAP1	ENSG00000137804	Nucleolar And Spindle Associated Protein 1	1.6951275	−0.52922344
MSH5-SAPCD1	ENSG00000255152	MSH5-SAPCD1 Readthrough (NMD Candidate)	1.6527612	−0.82210636
PTTG1	ENSG00000164611	Pituitary Tumor-Transforming 1	1.6005845	−0.7480469
FAM64A	ENSG00000129195	PICALM Interacting Mitotic Regulator	1.599808	−0.6055293
FANCI	ENSG00000140525	FA Complementation Group I	1.513165	−0.64473104
RNU6ATAC	ENSG00000221676	RNA, U6atac Small Nuclear (U12-Dependent Splicing)	1.5082383	−1.1415639
ITGA6	ENSG00000091409	Integrin Subunit Alpha 6	1.5013771	−0.5040817
EBNA1BP2	ENSG00000117395	EBNA1 Binding Protein 2	1.4999957	−0.57117844
LOC103021295	Entrez Gene:103021295	Uncharacterized LOC103021295	1.4921154	−1.6693618
PKI55	ENSG00000260804	Long Intergenic Non-Protein Coding RNA 1963	1.4866606	−0.7485008
NME1-NME2	ENSG00000011052	NME1-NME2 Readthrough	1.4815836	−1.7292972

CIT	ENSG00000122966	Citron Rho-Interacting Serine/Threonine Kinase	1.4401348	−0.5243051
TK1	ENSG00000167900	Thymidine Kinase 1	1.4356728	−1.0613041
SFN	ENSG00000175793	Stratifin	1.4122744	−0.5776167
NBPF25P	ENSG00000272150	NBPF Member 25, Pseudogene	1.4101803	−0.6656171
TIMM10	ENSG00000134809	Translocase Of Inner Mitochondrial Membrane 10	1.3986549	−0.71706486
TAF15	ENSG00000270647	TATA-Box Binding Protein Associated Factor 15	1.3981676	−0.6371021
AP2S1	ENSG00000042753	Adaptor Related Protein Complex 2 Subunit Sigma 1	1.3979273	−0.59551096
CCNE2	ENSG00000175305	Cyclin E	1.3833754	−0.58780575
RACGAP1	ENSG00000161800	Rac GTPase Activating Protein 1	1.3817348	−0.56450415
ASNS	ENSG00000070669	Asparagine Synthetase (Glutamine-Hydrolyzing)	1.3765383	−0.5224061
KIF18B	ENSG00000186185	Kinesin Family Member 18B	1.3718948	−0.6087103
EXOSC5	ENSG00000077348	Exosome Component 5	1.3604612	−0.52586985
IQGAP3	ENSG00000183856	IQ Motif Containing GTPase Activating Protein 3	1.3459537	−0.504246
HPRT1	ENSG00000165704	Hypoxanthine Phosphoribosyltransferase 1	1.3439608	−0.5530338
CDC20	ENSG00000117399	Cell Division Cycle 20	1.3311977	−1.0260725
FAM111B	ENSG00000189057	amily With Sequence Similarity 111 Member B	1.2990503	−0.78210616
CD151	ENSG00000177697	CD151 Molecule (Raph Blood Group)	1.2954721	−0.51596546
KNSTRN	ENSG00000128944	Kinetochore Localized Astrin (SPAG5) Binding Protein	1.288887	−0.5405283
NUF2	ENSG00000143228	NUF2, NDC80 Kinetochore Complex Component	1.2869172	−0.62100697
EFHD2	ENSG00000142634	EF-Hand Domain Family Member D2	1.2579031	−0.67007494
ITGB3BP	ENSG00000142856	ntegrin Subunit Beta 3 Binding Protein	1.2441993	−0.51290464
KIF2C	ENSG00000142945	Kinesin Family Member 2C	1.2170086	−0.68559694
HCFC1R1	ENSG00000103145	Host Cell Factor C1 Regulator 1	1.2103913	−0.72119904
DDIT3	ENSG00000175197	DNA Damage Inducible Transcript 3	1.201889	−1.4629793
TMPO-AS1	ENSG00000257167	TMPO Antisense RNA 1	1.1880759	−1.2009901
H3F3AP4	ENSG00000235655	H3 Histone, Family 3A, Pseudogene 4	1.1790832	−2.053651
TOB2P1	ENSG00000176933	Transducer Of ERBB2, 2 Pseudogene 1	1.17452	−0.6659017
PAGE1	ENSG00000068985	PAGE Family Member 1	1.1718446	−0.68176055
FABP5	ENSG00000164687	Fatty Acid Binding Protein 5	1.1528816	−0.85995007
LAS1L	ENSG00000001497	LAS1 Like, Ribosome Biogenesis Factor	1.1472235	−0.52020025
HAUS7	ENSG00000213397	HAUS Augmin Like Complex Subunit 7	1.1414261	−0.5131812
HJURP	ENSG00000123485	Holliday Junction Recognition Protein	1.1292663	−0.72957325
MCM6	ENSG00000076003	Minichromosome Maintenance Complex Component 6	1.119534	−0.8125825
POLA1	ENSG00000101868	DNA Polymerase Alpha 1, Catalytic Subunit	1.1152444	−0.931638
SERF2-C15ORF63	Entrez Gene:100529067	SERF2-C15orf63 Readthrough	1.1128592	−1.8926823
ATG3	ENSG00000144848	Autophagy Related 3	1.1122737	−0.60944414
E2F8	ENSG00000129173	E2F Transcription Factor 8	1.1110861	−0.56246686
PGK1	ENSG00000102144	Phosphoglycerate Kinase 1	1.1066875	−0.7288847
PSRC1	ENSG00000134222	Proline And Serine Rich Coiled-Coil 1	1.1043298	−0.75656366

LINC00941	ENSG00000235884	Long Intergenic Non-Protein Coding RNA 941	1.1040186	−0.6149818
SESN2	ENSG00000130766	Sestrin 2	1.097506	−1.0312753
RFESD	ENSG00000175449	Rieske Fe-S Domain Containing	1.095147	−0.56969726
YARS	ENSG00000134684	Tyrosyl-TRNA Synthetase	1.0863867	−0.8222084
SPA17	ENSG00000064199	Sperm Autoantigenic Protein 17	1.0854797	−0.6276326
KIF20A	ENSG00000112984	Kinesin Family Member 20A	1.0812011	−0.6162262
SKA2	ENSG00000182628	Spindle And Kinetochore Associated Complex Subunit 2	1.0760107	−0.691
PPP1R14B	ENSG00000173457	Protein Phosphatase 1 Regulatory Inhibitor Subunit 14B	1.0749106	−0.85723734
ANAPC15	ENSG00000173457	Anaphase Promoting Complex Subunit 15	1.0708952	−0.70507336
CDCA3	ENSG00000111665	Cell Division Cycle Associated 3	1.065907	−0.9970088
MTHFD2	ENSG00000065911	Methylenetetrahydrofolate Dehydrogenase (NADP+ Dependent) 2, Methenyltetrahydrofolate Cyclohydrolase	1.0653872	−0.7614031
RHEBL1	ENSG00000167550	RHEB Like 1	1.0574455	−0.50987935
MSH5	ENSG00000204410	MutS Homolog 5	1.0536244	−0.6192504
GARS	ENSG00000106105	Glycyl-TRNA Synthetase	1.0456743	−0.6545992
HIST1H2AH	ENSG00000274997	Histone Cluster 1 H2A Family Member H	1.0428671	−0.9570452
C21orf58	ENSG00000160298	Chromosome 21 Open Reading Frame 58	1.0424036	−0.51380897
HSPB11	ENSG00000081870	Heat Shock Protein Family B (Small) Member 11	1.0314069	−0.581501
MAGOHB	ENSG00000111196	Mago Homolog B, Exon Junction Complex Subunit	1.0288863	−0.71507716
NCAPD2	ENSG00000010292	Non-SMC Condensin I Complex Subunit D2	1.0174556	−0.5655904
RAC1	ENSG00000136238	Rac Family Small GTPase 1	1.012918	−0.5034852
PRC1	ENSG00000198901	Protein Regulator Of Cytokinesis 1	1.0084558	−0.5724373
NDC80	ENSG00000080986	NDC80, Kinetochore Complex Component	1.0041885	−0.77643156
PSIP1	ENSG00000164985	PC4 And SFRS1 Interacting Protein 1	1.0004644	−0.6289239

Table S2. Univariate analysis of variables associated with E2F1 activity in TCGA LIHC cohort.

Variable	Total	E2F1_High (%)	OR [95%CI]	RR [95%CI]	*p*-value
Sex					
male	251	114 (45%)	0.57 [0.36–0.88]	0.76 [0.62–0.93]	0.0149
female	121	72 (60%)	1	1	
Age					
55 >	244	113 (46%)	0.65 [0.42–1.00]	0.81 [0.66–0.99]	0.0635
55 ≤	128	73 (57%)	1	1	
65 >	135	60 (44%)	0.70 [0.46–1.08]	0.84 [0.67–1.05]	0.1311
65 ≤	237	126 (53%)	1	1	
Tumor stage					
I–II	258	117 (45%)	0.46 [0.28–0.75]	0.70 [0.57–0.86]	0.0027
III–	90	58 (64%)	1	1	

OR = Odds Ratio; RR = Rerative Risk; 95%CI = 95% confidence intervals; *p*-value = Chi-Square Pearson *p*-value.

Table S3. Univariate analysis of variables associated with KIF gene set (KIF2C, KIF18B, and KIF20A) activity in TCGA LIHC cohort.

Variable	Total	E2F1_High (%)	OR [95%CI]	RR [95%CI]	*p*-value
Sex					
male	251	116 (46%)	0.63 [0.40–0.97]	0.80 [0.65–0.98]	0.0464
female	121	70 (58%)	1	1	
Age					
55 >	247	115 (47%)	0.66 [0.43–1.02]	0.82 [0.67–1.00]	0.0790
55 ≤	125	71 (57%)	1	1	
65 >	138	57 (41%)	0.57 [0.37–0.88]	0.75 [0.60–0.94]	0.0136
65 ≤	234	129 (55%)	1	1	
Tumor stage					
I–II	258	118 (46%)	0.47 [0.28–0.76]	0.71 [0.58–0.87]	0.0033
III–	90	58 (64%)	1	1	

OR = Odds Ratio; RR = Rerative Risk; 95%CI = 95% confidence intervals; *p*-value = Chi-Square Pearson *p*-value.

Table S4. List of the primers' sequences.

Gene	Primers	Sequences (5′-3′)
ACTB	F	5′-GCA AAG ACC TGT ACG CCA AC-3′
	R	5′-GGA GCA ATG ATC TTG ATC TTC A-3′
E2F1	F	5′-CCA GGA GGT CAC TTC TGA GG-3′
	R	5′-ACA ACA GCG GTT CTT GCT C-3′
EMC7	F	5′-ACA TGA GAC GGG AAA TGG AG-3′
	R	5′-CCC CAC TTT TGC CTG TTT TA-3′
KIF18A	F	5′-ATG TGG ACA GGA GGA CTT GG-3′
	R	5′-GAA GAA GGA TCA AGC CGT TG-3′
KIF18B	F	5′-TTC AGA GTC AAT CCC TGT GC-3′
	R	5′-GAT TCC CAG GGT GTG CAG-3′
KIF20A	F	5′-CAT CTT AAT CAA ACA GGA CCA GA-3′
	R	5′-AGA AAC CTG GCC TTG GAG TT-3′
KIF23	F	5′-CCA AAT GGT AGT CGA AAA CG-3′
	R	5′-CTC TCA TCT CCA CAG CCA CA-3′
KIF2C	F	5′-TCC AGG CAA TTT ATC CAA GG-3′
	R	5′-CCA GTC TGG TCC TTG CTG TA-3′
KNSTRN	F	5′-TGT CAG AAA AGG CTA CAA ACC A-3′
	R	5′-GGG TCA GGT CCT TCA GCT CT-3′
MCT4	F	5′-CTT CGG GAG GCA AAC TCC T-3′
	R	5′-TGG GCT TCT TCC TAA TGC AG-3′
NDC80	F	5′-TGA AAA ATG TGC CAG TGA GC-3′
	R	5′-GTT GGT ATT CCC GCT GAA CA-3′

NUASP1	F	5′-GCT TCT ACT CCC ATC AGC CA-3′
	R	5′-CTG AAA ACC TGA CAC CCG TT-3′
NUF2	F	5′-AAG AGG TGC TGT CTA TGA ACG A-3′
	R	5′-AAA TAT TTC CTG GGA CTT CAG TTT-3′
PRC1	F	5′-GCA AGA TCA AGA ACT GTG CG-3′
	R	5′-TCA CGC CTA GAA GCC TTT GT-3′
STMN1	F	5′-AAG ACG CAA GTC CCA TGA AG-3′
	R	5′-GCT TCC ATT TTG TGG GTC AG-3′

© 2019 by the authors. Licensee MDPI, Basel, Switzerland. This article is an open access article distributed under the terms and conditions of the Creative Commons Attribution (CC BY) license (http://creativecommons.org/licenses/by/4.0/).
